# Nanodiamonds Interact with Primary Human Macrophages and Dendritic Cells Evoking a Vigorous Interferon Response

**DOI:** 10.1021/acsnano.4c18108

**Published:** 2025-05-14

**Authors:** Tomas Malina, Jasreen Kaur, Sebastin Martin, Audrey Gallud, Shintaro Katayama, Arianna Gazzi, Marco Orecchioni, Martin Petr, Martin Šrejber, Lars Haag, Bejan Hamawandi, Muhammet S. Toprak, Juha Kere, Lucia Gemma Delogu, Bengt Fadeel

**Affiliations:** † Institute of Environmental Medicine, Division of Molecular Toxicology, 27106 Karolinska Institutet, 171 77 Stockholm, Sweden; ‡ Nanotechnology Centre, Centre for Energy and Environmental Technologies, VSB-Technical University of Ostrava, 708 00 Ostrava Poruba, Czech Republic; § Regional Centre of Advanced Technologies and Materials, Czech Advanced Technology and Research Institute (CATRIN), Palacký University, 772 00 Olomouc, Czech Republic; ∥ Department of Biosciences and Nutrition, Karolinska Institutet, 148 13 Huddinge, Sweden; ⊥ Department of Biomedical Sciences, 9308 University of Padua, Padua 351 29, Italy; # La Jolla Institute for Immunology, San Diego, California 92037, United States; ∇ Immunology Center of Georgia, 1421 Augusta University, Augusta, Georgia 30912, United States; ○ Department of Laboratory Medicine, Karolinska Institutet, 141 52 Huddinge, Sweden; ◆ Department of Applied Physics, KTH-Royal Institute of Technology, 106 91 Stockholm, Sweden; ¶ Stem Cells and Metabolism Research Program (STEMM), University of Helsinki, 00290 Helsinki, Finland; †† Department of Biological Sciences, Khalifa University of Science and Technology, P.O. Box 127788 Abu Dhabi, United Arab Emirates

**Keywords:** autophagy, dendritic cells, interferon, macrophages, nanodiamonds

## Abstract

Nanodiamonds (NDs) display several attractive features rendering them useful for medical applications such as drug delivery. However, the interactions between NDs and the immune system remain poorly understood. Here, we investigated amino-, carboxyl-, and poly­(ethylene glycol) (PEG)-terminated NDs with respect to primary human immune cells. We applied cytometry by time-of-flight (CyToF) to assess the impact on peripheral blood mononuclear cells at the single-cell level, and observed an expansion of plasmacytoid dendritic cells (pDCs) which are critically involved in antiviral responses. Subsequent experiments demonstrated that the NDs were actively internalized, leading to a vigorous type I interferon response involving endosomal Toll-like receptors. ND-NH_2_ and ND-COOH were more potent than ND-PEG, as evidenced by using TLR reporter cell lines. Computational studies demonstrated that NDs interacted with the ligand-binding domains of TLR7 and TLR9 with high affinity though this was less pronounced for ND-PEG. NDs with varying surface functionalities were also readily taken up by macrophages. To gain further insight, we performed RNA sequencing of a monocyte-like cell line exposed to NDs, and found that the phagosome maturation pathway was significantly affected. Indeed, evidence for lysosomal hyperacidification was obtained in dendritic cells and macrophages exposed to NDs. Moreover, using a reporter cell line, NDs were found to impinge on autophagic flux. However, NDs did not affect viability of any of the cell types studied. This study has shown that NDs subvert dendritic cells leading to an antiviral-like immune response. This has implications not only for drug delivery but also for anticancer vaccines using NDs.

## Graphical abstract

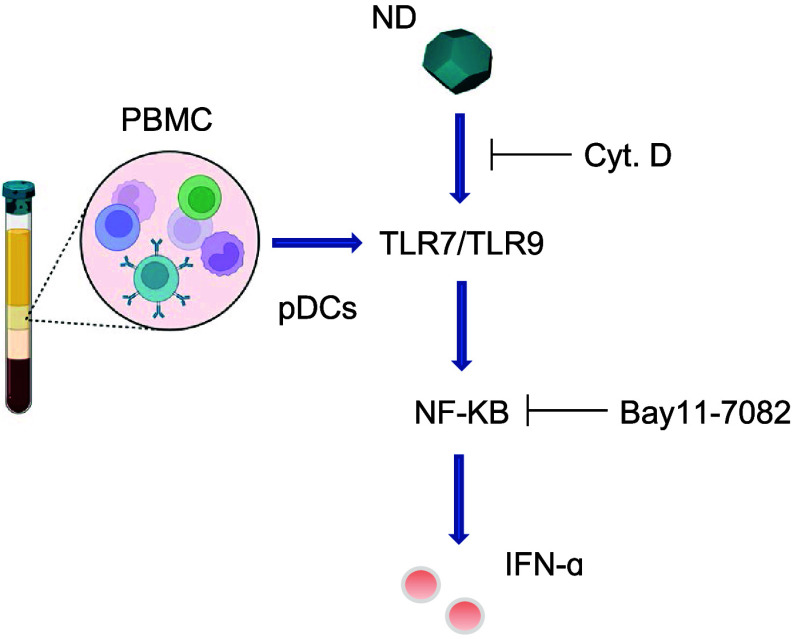

## Introduction

Nanodiamonds (NDs) exhibit several interesting and useful features such as high biocompatibility, tunability of surface properties, and versatility with respect to their cargo.^1,2^ Moreover, some NDs are so small that they seem to violate the boundaries between particles and molecules.^3^ This is not merely of academic interest as nanoparticles could potentially be harnessed to modulate biological systems.^4^ For instance, gold nanoparticles have demonstrated intrinsic therapeutic potential.^5,6^ Moreover, nanoparticles have shown promise as adjuvants to boost the effect of vaccines.^4^ The unique properties of the faceted surfaces of NDs have been exploited for drug delivery in several preclinical models.^7^ For instance, Chow et al. demonstrated that doxorubicin-conjugated NDs could overcome chemoresistance,^8^ and subsequent studies confirmed the suitability of NDs as a drug delivery platform.^9,10^

Safety assessment is required in order to shepherd novel nanomaterials into the clinic.^11^ Early work indicated that NDs displayed cytotoxicity in serum-free cell culture medium while no toxicity was observed in serum-containing medium,^12^ and it has been suggested, using a cancer cell line, that NDs are actively internalized via endocytosis.^13^ NDs were subsequently found to be neither cytotoxic nor genotoxic toward a wide range of cell lines even at doses as high as 250 μg/mL.^14^ It is also notable that NDs are well-tolerated in rodents and nonhuman primates at clinically relevant doses.^15^ Moreover, recent *in vitro* and *in vivo* studies conducted in the frame of the EU-funded FP7-NANOSOLUTIONS project have shown NDs to be remarkably biocompatible in comparison to a wide range of other nanomaterials.^16,17^ These studies,^16^ and related work by other investigators,^18^ have thus shown that NDs do not affect cell viability of THP-1 cells (a commonly used model of monocytes-macrophages)^19^ or primary human monocyte-derived macrophages (up to 50–100 μg/mL). However, there are few if any studies on the interactions of NDs with primary dendritic cells (DCs) even though previous studies have demonstrated that other carbon-based nanomaterials could influence DCs.^20–22^ DCs are specialized in the uptake, processing, and presentation of antigen thereby promoting antimicrobial immune responses. It has been shown that carboxylic acid modified NDs (ND-COOH) elicit a modest dose- and time-dependent effect toward monocytes whereas amino-functionalized NDs (ND-NH_2_) were found to be noncytotoxic (at concentrations up to 100 μg/mL).^23^ The latter study demonstrated that ND-COOH provoked more pronounced changes in gene expression in peripheral blood mononuclear cells (PBMCs) (50 μg/mL) when compared to ND-NH_2_. The surface properties of NDs may thus play an important role for immune cell interactions. Using THP-1 cells as a model, other investigators proposed that the sp^3^/sp^2^ carbon ratio is a key determinant of the toxicity of NDs.^24^ Notwithstanding, a comprehensive understanding of the impact of NDs on primary human immune-competent cells is lacking, especially at concentrations that do not elicit overt cell death. In particular, the possible impact on DCs remains poorly understood. DCs serve as sentinels of the immune system, providing a link between innate and adaptive immunity. As such, these cells are a key target for immunotherapy.^25^ In the present study, we investigated NDs with different surface functionalities (ND-NH_2_, ND-COOH, and ND-PEG) (Scheme S1) with respect to their interactions with PBMCs using single-cell mass cytometry or CyToF, a high-dimensional method uniquely suited for the profiling of immune cell responses to nanomaterials.^26,27^ We also investigated the impact of NDs on primary monocytes as well as monocyte-derived DCs and monocyte-derived macrophages. NDs were internalized by these cells without signs of cytotoxicity. Furthermore, NDs were found to influence DC maturation, and evidence was provided, based on transcriptomics analysis and functional assays, for the impairment of phagosomal maturation in ND-exposed cells. Finally, we showed for the first time that NDs are sensed by plasmacytoid dendritic cells (pDCs) via Toll-like receptors (TLR7 and TLR9) leading to a type I interferon response. These results were supported by computational studies showing high binding affinities between NDs and TLR7/TLR9. These findings are relevant for biomedical applications such as drug delivery and anticancer vaccines.^28^

## Results and Discussion

### Characterization of Surface Modified NDs

NDs with different surface modifications (ND-NH_2_, ND-COOH, and ND-PEG) were described previously.^16^ Transmission electron microscopy (TEM) images showing the size and shape of the NDs are displayed in Figure 1A. The three samples thus contain very small crystalline particles that tend to cluster together in larger agglomerates (Figure 1A). Moreover, high resolution (HR) TEM of the ND-COOH sample confirmed the dominant crystalline plane indexed for the (111) plane of the diamond lattice (Figure 1B).

**Figure 1:**
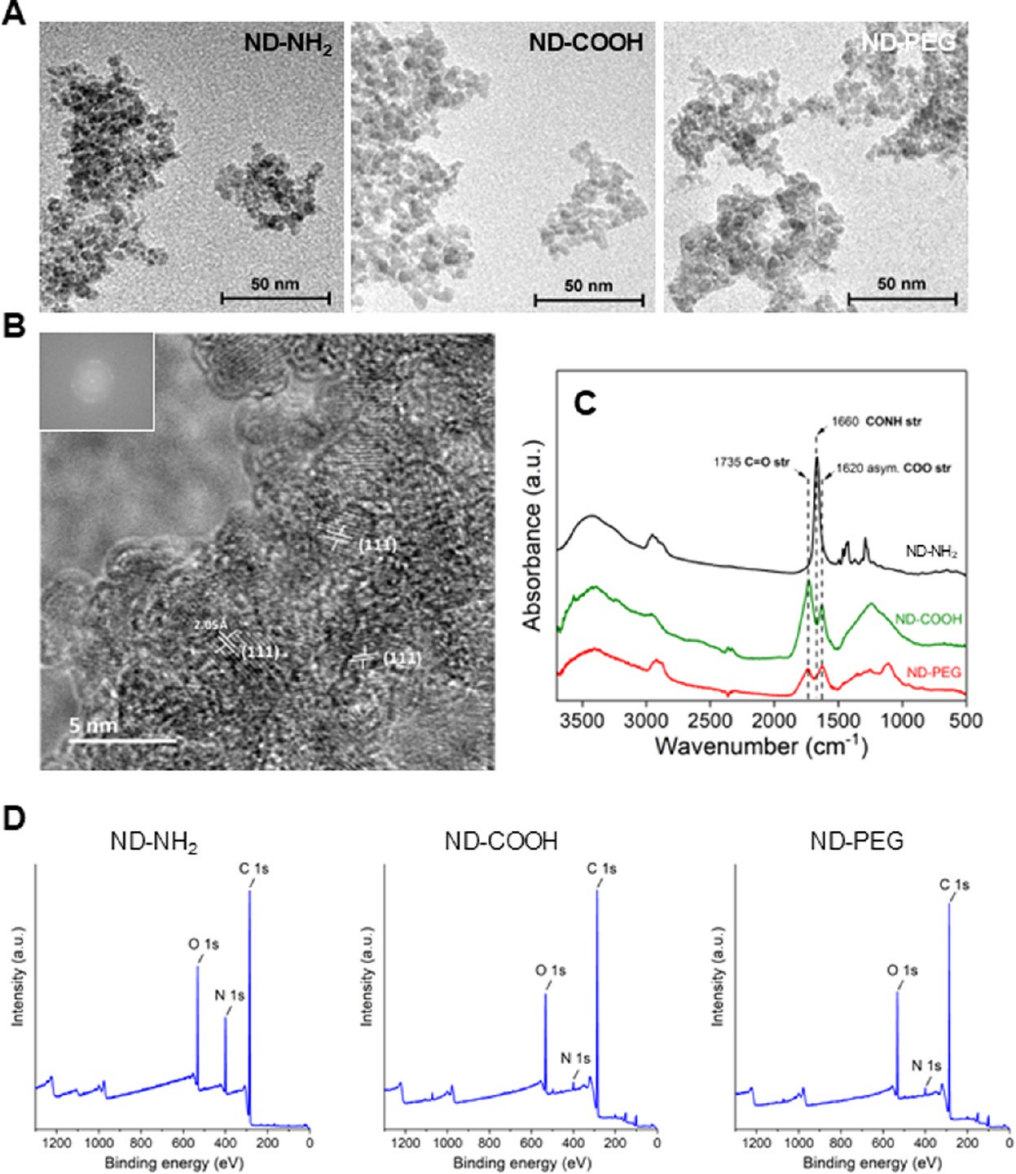
ND characterization. (A) TEM images of ND-NH_2_, ND-COOH, and ND-PEG. Scale: 50 nm. (B) HR-TEM of ND-COOH revealing (111) diamond lattice lines. Scale: 5 nm. (C, D) Characterization of surface functionalization of NDs with spectroscopy techniques. (C) FTIR spectra of ND-NH_2_, ND-COOH, and ND-PEG. (D) XPS survey spectra. For high-resolution fitted spectra of C 1s and atomic composition, refer to Figure S1.

The surface functionalization of the NDs was verified using two different spectroscopy techniques. Fourier Transform Infrared (FTIR) spectroscopy revealed three important bands in the ND samples. Two distinctive peaks at 1735 and 1620 cm^–1^, corresponding to the C = O and asymmetric COO stretching, were found in ND-COOH and ND-PEG, while they were absent in the ND-NH_2_ sample (Figure 1C). On the other hand, a shifted peak at 1660 cm^–1^ was identified in the ND-NH_2_ sample representing the amide bond obtained by replacing the −OH in carboxyl by the −NH_2_ group (Figure 1C). NDs were also evaluated by means of X-ray photoelectron spectroscopy (XPS). Survey spectra demonstrated that a higher atomic percentage of nitrogen could be detected in the ND-NH_2_ sample (13.5%) compared to the marginal values detected in ND-COOH and ND-PEG (2 and 2.1%) (Figure 1D). Furthermore, high-resolution C 1s spectra revealed that the dominant surface functionalities in ND-COOH and ND-PEG were hydroxyl (C–O), carbonyl (C=O) and carboxyl (O=C–O) groups (Figure S1). On the other hand, C–N and C=N were the two distinctive groups fitted in the high-resolution spectra of C 1s of ND-NH_2_, as expected with the increased nitrogen content in this sample (Figure S1). Taken together, FTIR and XPS confirmed the surface modifications of the three NDs.

To further characterize the samples, DLS analysis was performed in both Milli-Q water and RPMI-1640 medium supplemented with 10% fetal bovine serum (FBS). In water, the average hydrodynamic diameter for ND-COOH and ND-PEG was around 150 nm, while for the ND-NH_2_ the diameter was around 300 nm (Figure S2A). In cell culture medium, no difference between materials was observed, as all three materials displayed a diameter of around 150 nm (Figure S2A). ζ-potential measurements confirmed the differences between the surface functionalities insofar as ND-NH_2_ was the only sample with a positive value (+5 mV), while ND-COOH and ND-PEG showed negative values (−40 and −33 mV, respectively) (Figure S2B). In cell culture medium, the ζ-potential of all three NDs was “equalized” with values around – 10 mV (Figure S2B). This result is expected when nanoparticles are immersed in serum-containing medium.^29^ The NDs were also evaluated for possible endotoxin contamination. No contamination was observed in any of the ND samples (Figure S3).

### No Toxicity in Primary Human Immune Cells

To comprehensively investigate the impact of NDs on immune cells, we applied single-cell mass cytometry or cytometry by time-of-flight (CyToF), a method that allows for the discrimination of 15 distinct subpopulations of PBMCs from healthy adult donors.^30^ First, biocompatibility of the NDs was evaluated using the LDH release assay. No toxicity was observed when PBMCs were exposed to ND-COOH, ND-NH_2_, or ND-PEG up to 100 μg/mL for 24 h (Figure 2A,B). The results were corroborated using the Alamar Blue assay (data not shown). We selected 20 μg/mL for subsequent CyToF experiments. The cells were exposed for 24 h and samples were processed as described in the Experimental section, and *t*-SNE (*t*-distributed stochastic neighbor embedding), an unsupervised dimensionality reduction technique, was used to display the data.^31^ We thus identified all the major immune cell populations in control and ND-exposed samples (Figure 3A). Bacterial lipopolysaccharide (LPS) was included as a positive control for immune cell activation.^32^ CyToF revealed that ND-COOH incubation led to a decrease in the number of classical monocytes (denoted in green in the viSNE plot) when compared to control, while ND-NH_2_ and ND-PEG did not significantly affect these cells. Some changes were also seen with respect to natural killer (NK) cells (denoted in gray). Moreover, all three NDs increased the number of pDCs (denoted in pink in the viSNE plot) (Figure 3A). Plasmacytoid DCs are a unique subset of DCs with crucial roles in immunity particularly in antiviral responses.^33^ We also utilized Cell-ID Intercalator-Ir to distinguish live cells from dead cells. The data showed that NDs were well-tolerated by all subpopulations of PBMCs (at the tested concentration and time-point) irrespective of the surface functionalization (Figure 3B–F).

**Figure 2:**
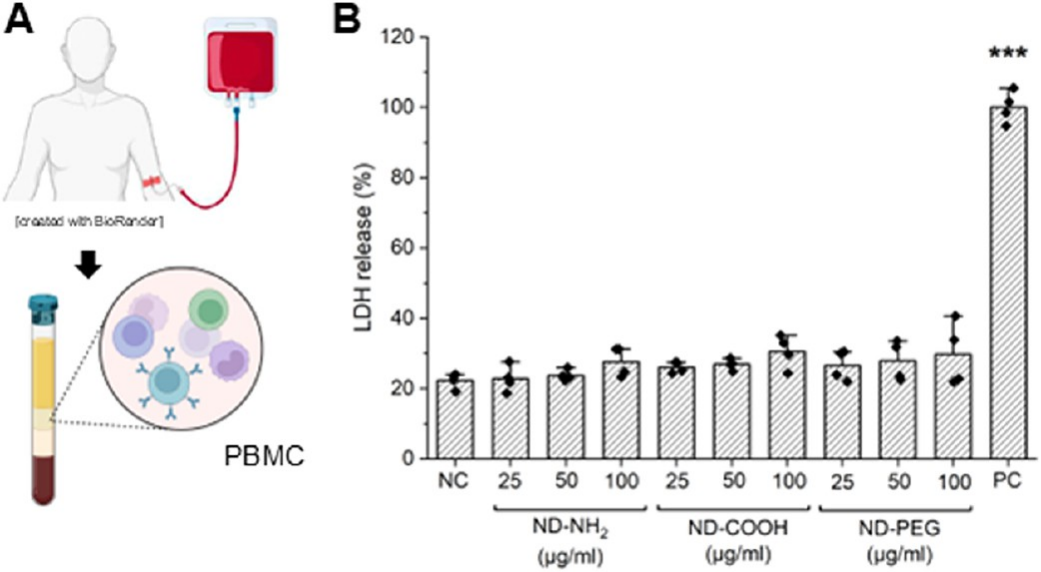
NDs are noncytotoxic for PBMCs. PBMCs isolated from three independent human donors (A) were exposed for 24 h to the different NDs at the indicated concentrations (B). Cell viability was monitored using the LDH release assay. PC, positive control. One-way Anova with Dunnett’s post hoc test was applied. ***p ≤ 0.001. (A) was generated by using BioRender.com under an academic license.

**Figure 3:**
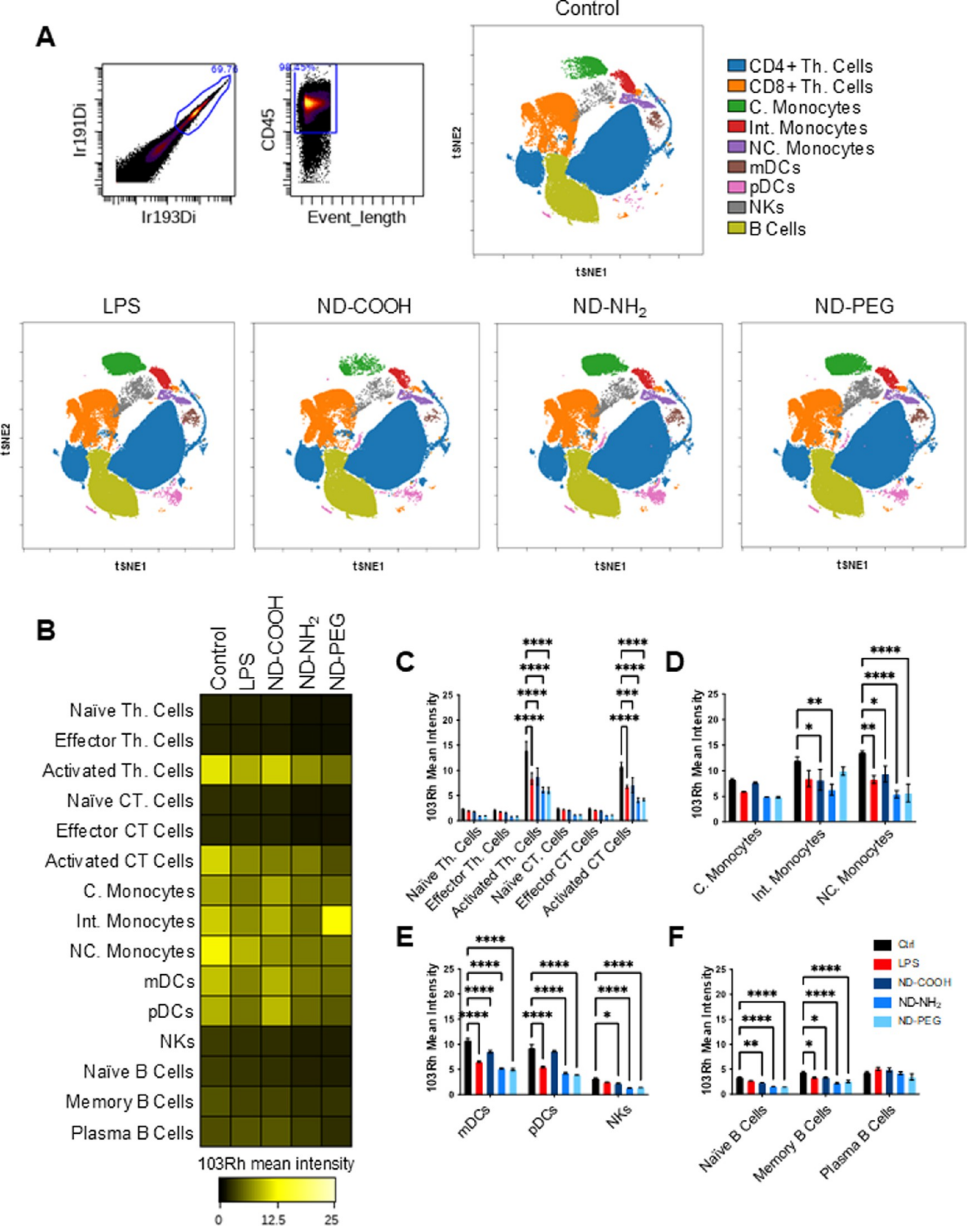
Single-cell profiling of PBMCs. PBMCs were exposed to ND-COOH, ND-NH_2_, and ND-PEG (20 μg/mL) for 24 h or left untreated (control). LPS (0.1 μg/mL) was used as a positive control for immune cell activation. (A) viSNE analysis displays the single-cell subpopulations. Uncalled cells are not shown. (B) Heatmap of mean marker expression for all gated immune subpopulations. (C) Histograms of rhodium mean marker expression ratio for gated T cell populations, (D) monocyte cell populations, (E) DC and NK cell populations, and (F) B cell populations. The latter data are shown as mean values ± SD of four independent samples. Heatmaps and viSNE plots were generated from the concatenated files. Two-way Anova with Tukey’s multiple comparison versus control was used. *p < 0.05, **p < 0.01, ***p < 0.001, ****p < 0.0001.

### NDs Trigger Plasmacytoid DC Expansion

Myeloid DCs (mDCs) and plasmacytoid DCs (pDCs) are two distinct subsets of DCs.^33^ The former are primarily involved in antigen presentation, while pDCs are known for their capacity to produce type I interferons (IFNs) in response to viral infections. Indeed, pDCs constitute only about 0.5% of PBMCs in healthy individuals, but account for 95% of the IFN produced by PBMCs in response to viruses (discussed below). Intrigued by the single-cell mass cytometry findings, we proceeded to analyze surface markers of mDCs (CD11c, HLA-DR) and pDCs (CD123, CD303) by flow cytometry following exposure of PBMCs to ND-NH_2_, ND-COOH, and ND-PEG (25 μg/mL). No differences were noted in the percentage of CD11c-positive and HLA-DR positive cells (Figure S4A,C) whereas the number of both CD123-positive and CD303-positive cells was significantly increased following exposure to all three NDs compared to the untreated control (Figure S4E,F). Moreover, the expression of cell surface markers as determined by mean fluorescence intensity (MFI) of the positively stained population was significantly higher for mDC markers (Figure S4B,D) but not for pDC markers (data not shown). Hence, interactions of NDs with PBMCs resulted in the activation of mDCs as well as in increased numbers of pDCs irrespective of the surface modifications of NDs.

We then asked whether the NDs were taken up by PBMCs. To this end, PBMCs were exposed to NDs (25 μg/mL) for 24 h and uptake was monitored based on light scattering.^34^ Uptake was observed for all three NDs, and this was blocked by cytochalasin D (10 μM), a known inhibitor of actin polymerization^32^ (Figure 4A). Quantification of the results suggested that there was a slightly higher uptake of ND-NH_2_ when compared to ND-COOH and ND-PEG (Figure 4B), and side scatter values were higher for ND-NH_2_ indicating that more ND-NH_2_ were internalized per cell as compared to the other NDs (Figure S6A). However, uptake appeared to be restricted to a subpopulation (∼10%) of the PBMCs (Figure 4A). We decided to stain the PBMCs with antibodies against HLA-DR, a surface marker of antigen-presenting cells (Figure S5), and repeated the analysis of ND uptake for the latter population. The results revealed that HLA-DR-positive cells internalized the NDs (∼50% of the cells were positive for uptake, with similar results observed for all three NDs), and uptake was diminished in the presence of cytochalasin D (Figure 4C and S6B). Overall, these results provide evidence that NDs are preferentially taken up by antigen-presenting cells.

**Figure 4:**
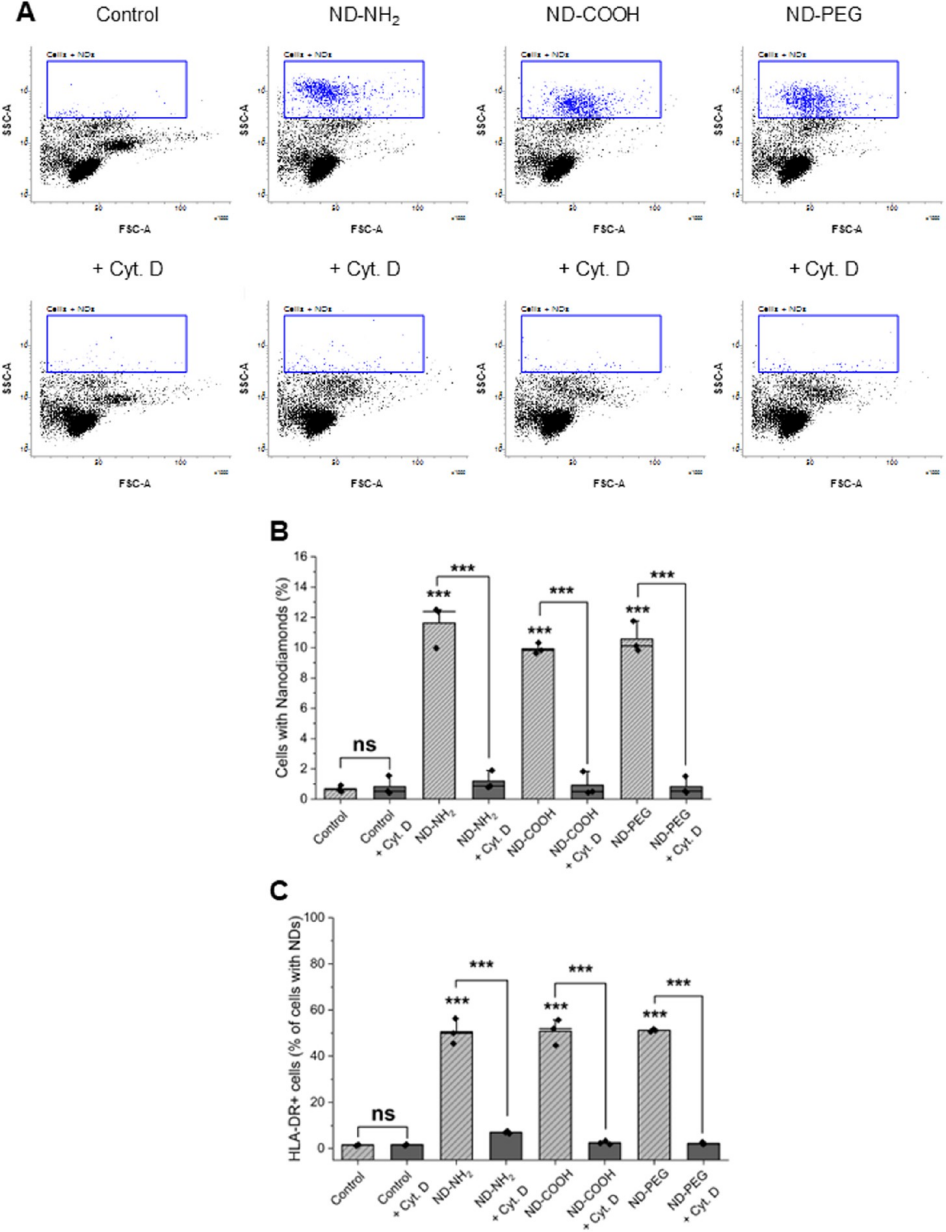
NDs are endocytosed by an HLA-DR-positive subpopulation of PBMCs. (A) Flow cytometric analysis of light scatter intensity is presented for PBMCs exposed to ND-NH_2_, ND-COOH, and ND-PEG for 24 h in the presence or absence of cytochalasin D (10 μM). (B) Quantification of the results. (C) Cells were stained with antibodies against HLA-DR, a surface marker expressed by antigen-presenting cells, and the analysis was performed on the gated population; refer to Figure S5 for gating, and Figure S6 for mean fluorescence intensity (MFI) values. Results displayed in panel (B, C) are shown as mean values ± SD obtained using cells from three individual human donors. Student’s *t*-test was applied to determine statistical significance. ***p < 0.001.

### NDs Trigger Activation/Maturation of DCs

To further investigate the impact of NDs on specific immune cell populations, we studied human monocyte-derived dendritic cells (MDDCs) and human monocyte-derived macrophages (HMDMs). Both cell populations can be derived from primary CD14-positive cells (monocytes) isolated from PBMCs.^35,36^ First, we used a standard protocol for the *ex vivo* differentiation of MDDCs, and flow cytometry was applied to confirm the expression of the cell surface markers, HLA-DR, CD11c, CD86, CD83, CD80, and CD40 (data not shown). Then, we assessed the interaction of ND-NH_2_, ND-COOH, and ND-PEG (25 μg/mL) with these cells. Scanning electron microscopy (SEM) demonstrated the presence of clusters/agglomerates of NDs on the cell membrane (Figure 5A) while TEM analysis revealed the presence of NDs in the cytosol (Figure S7). SEM also disclosed that MDDCs seemed to “probe” agglomerates of NDs using long and slender dendrites (Figure S8). No cytotoxicity was observed for any NDs up to 100 μg/mL (Figure 5B). We also found that the exposure to NDs resulted in DC activation and maturation, as evidenced by the upregulation of CD80, CD86, CD40, and CD83. Hence, all three NDs increased the expression of CD40, CD80, and CD86 in MDDCs (Figure S9A–C). For CD83, the most characteristic marker of DC maturation, expression was significantly elevated for all NDs, but ND-NH_2_ was found to elicit the highest response (Figure S9D). We also examined the expression of cell surface markers commonly expressed on DCs in CD14-positive monocytes (i.e., the precursors of MDDCs) and observed that the number of cells expressing CD86, HLA-DR, and CD11c was elevated following exposure for 24 h to NDs (25 μg/mL) (Figure S10A–C). Hence, the NDs promoted the differentiation of primary human monocytes into DC-like cells, as well as activation and maturation of DCs. Next, HMDMs were generated *ex vivo* from CD14-positive monocytes, and these cells were exposed for 24 h to ND-COOH, ND-NH_2_, or ND-PEG (25 μg/mL). The uptake of NDs was quantified by using flow cytometry. Prominent uptake was observed (∼90% of the cells were positive for uptake for all three NDs), and this was blocked in the presence of cytochalasin D, suggesting active endocytosis (Figure 6A,B). TEM confirmed an abundant uptake of all three NDs, and NDs were observed as agglomerates in intracellular vesicles (endosomes-lysosomes) (Figure 7A). No cytotoxicity was noted (Figure 7B). Macrophage uptake of NDs is neither unprecedented nor surprising, as these cells are professional phagocytes. However, previous studies typically applied cell lines as a model,^18,37^ whereas the present study was conducted using primary macrophages. Nevertheless, it must be noted that macrophages are present in tissues, not in peripheral blood. Instead, using PBMCs isolated from human donors, we showed that ND-NH_2_, ND-COOH, and ND-PEG are internalized by HLA-DR-positive (antigen-presenting) cells, and the uptake of NDs was also confirmed in primary human MDDCs.

**Figure 5:**
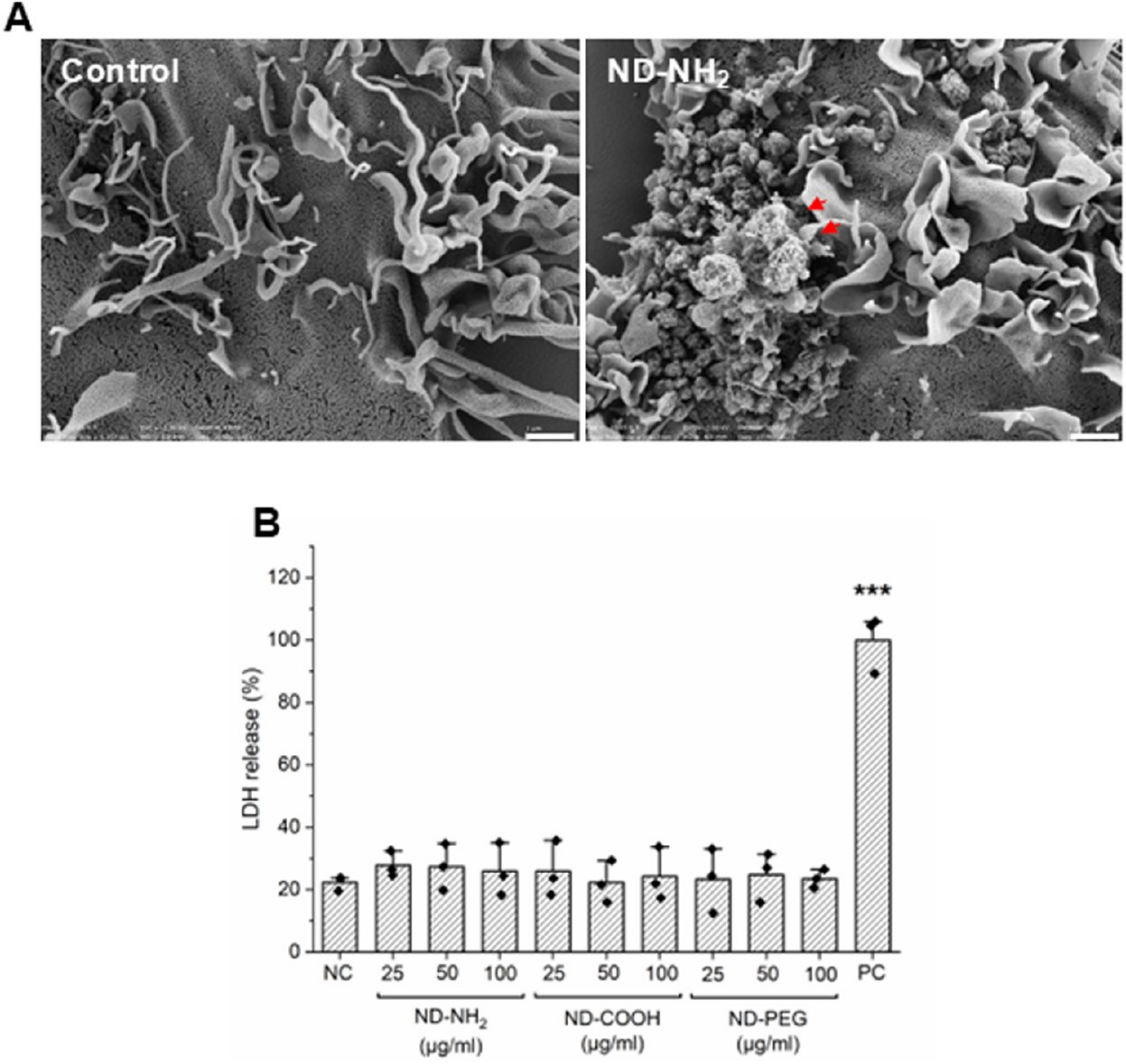
ND interact with MDDCs. (A) SEM images of MDDCs following exposure for 24 h to ND-NH_2_ (25 μg/mL) versus control. Red arrows mark agglomerates of NDs on the cell surface. Scale bars: 1 μm. (B) No loss of cell viability was observed using the LDH assay. Lysed cells were included as a positive control (PC). One-way Anova with Dunnett’s post hoc test was applied to determine statistical significance. ***p ≤ 0.001.

**Figure 6:**
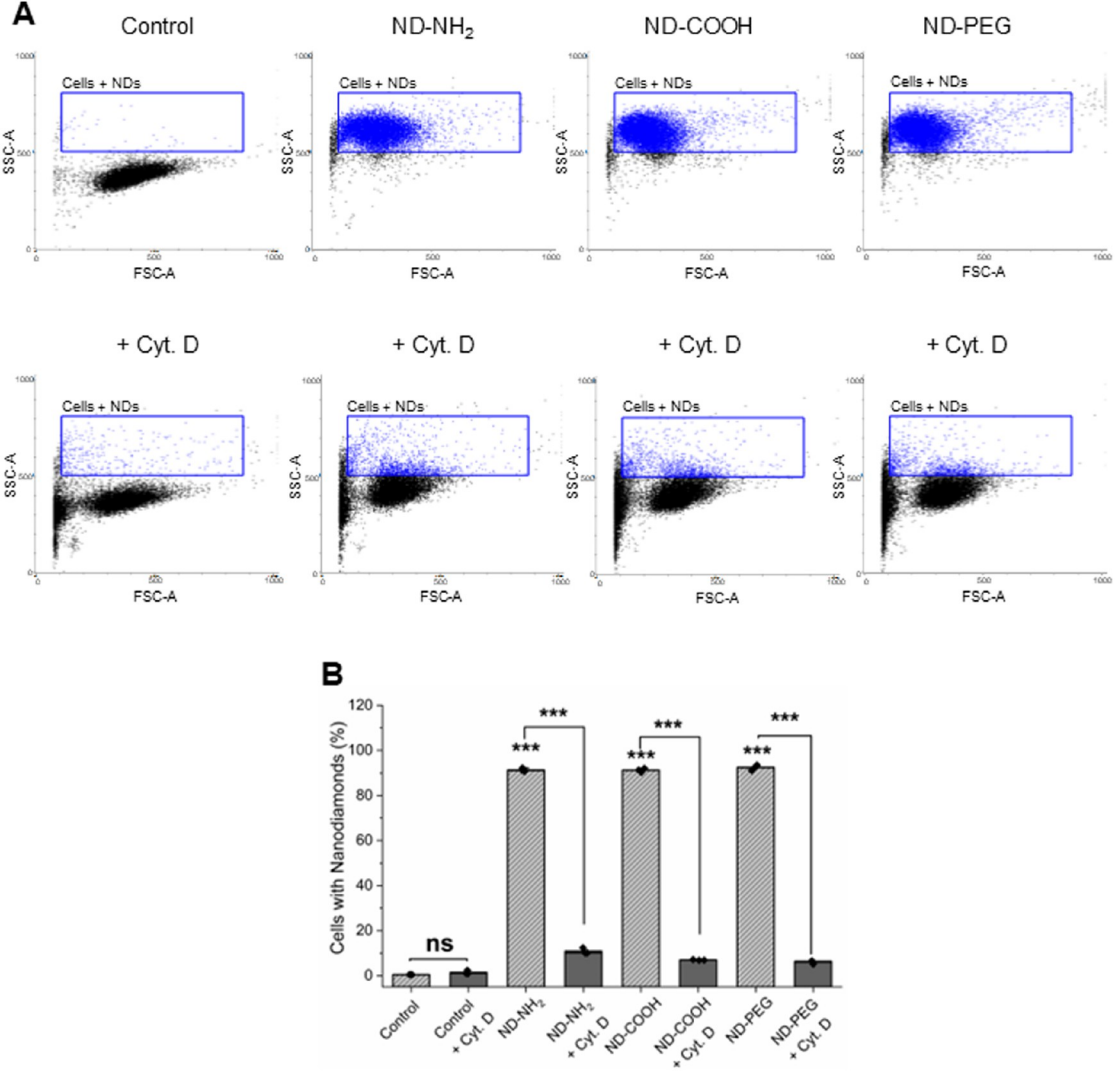
NDs are internalized by HMDMs through endocytosis. (A) Flow cytometric analysis of light scatter intensity for cells exposed to ND-NH_2_, ND-COOH, and ND-PEG for 24 h in the presence or absence of cytochalasin D (10 μM). (B) Quantification of the results. Results are shown as mean values ± SD using cells from three individual donors. Student’s *t*-test was applied to determine statistical significance. ***p < 0.001.

**Figure 7:**
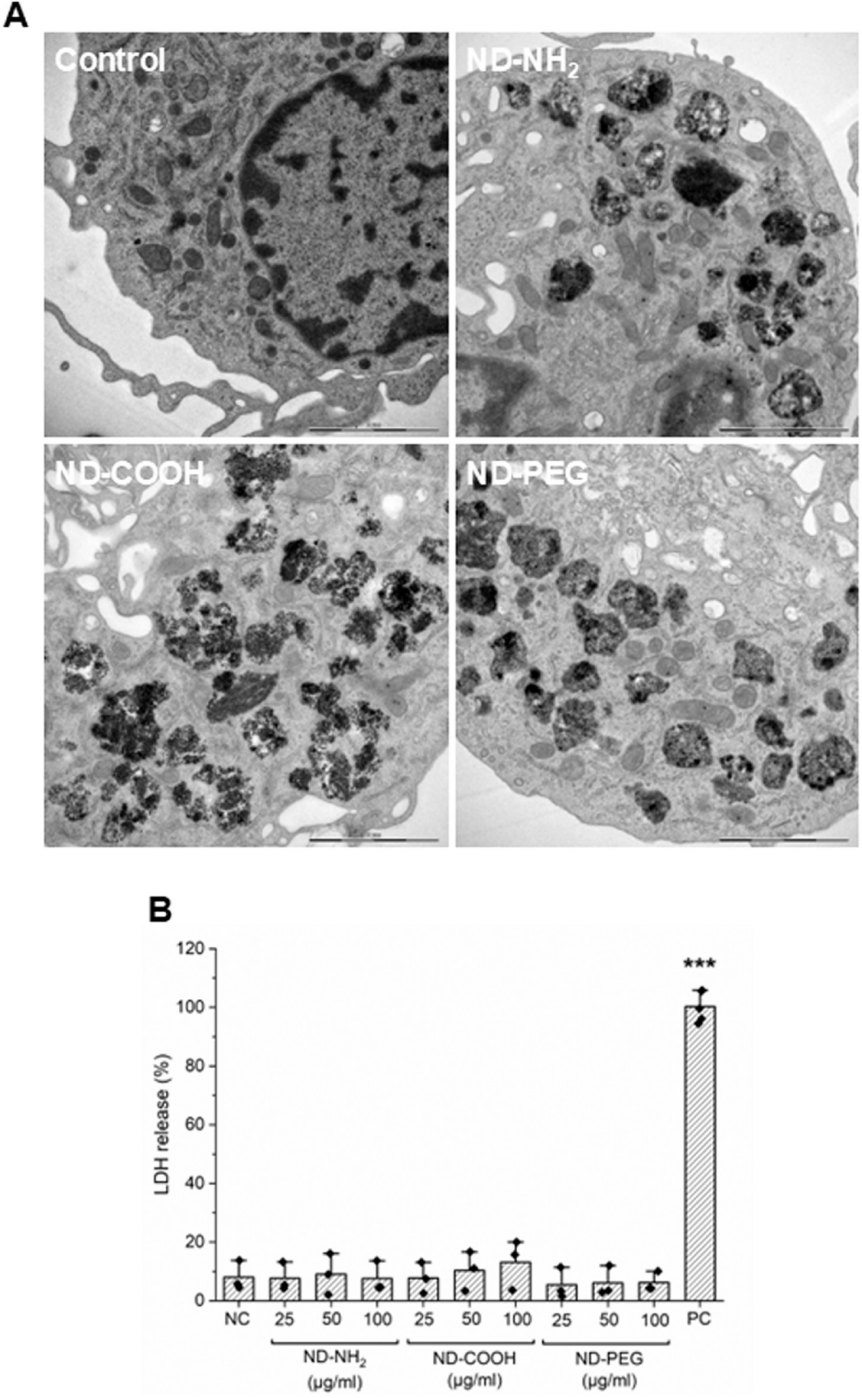
NDs are taken up by HMDMs. (A) TEM images of HMDMs exposed for 24 h to ND-NH_2_, ND-COOH, and ND-PEG (25 μg/mL). Scale bars: 2 μm. (B) No loss of cell viability was observed using the LDH release assay. Lysed cells were included as a positive control (PC). One-way Anova with Dunnett’s post hoc test was applied. ***p ≤ 0.001.

### NDs Impinge on Phagosome Maturation

To gain further insight into the impact of NDs at the molecular level, we performed RNA sequencing. To this end, the human monocyte-like THP-1 cell line was selected as a model, as cell lines are known to be a robust model for transcriptomics studies.^38,39^ We previously reported that these NDs are noncytotoxic toward THP-1 cells up to 100 μg/mL.^16^ To confirm cellular uptake of the NDs, we exposed THP-1 cells to NDs (50 μg/mL) and cells were analyzed by TEM. Clusters (agglomorates) of NDs were readily identified in the cells (Figure S11). Then, THP-1 cells (undifferentiated) were exposed to ND-NH_2_, ND-COOH, and ND-PEG (20 μg/mL) and RNA sequencing was performed as described in the Experimental section. We identified 2743, 1242, and 776 differentially expressed genes (DEGs), respectively (Figure 8A). The majority of these genes were downregulated (Supporting Tables S1, S2, S3, and S4). Then, pathway enrichment analysis was performed on the 526 common DEGs (Figure 8A) using the Ingenuity Pathway Analysis (IPA) software.^40^ The results were displayed by means of hierarchical clustering of the canonical pathways (Figure 8B). This cross comparison between the different exposures showed that the most significant pathway associated with the deregulated DEGs was the “phagosome maturation” pathway (*p* = 4.66 × 10^–12^ for ND-NH_2_; *p* = 2.91 × 10^–05^ for ND-COOH; *p* = 6.38 × 10^–05^ for ND-PEG). The genes significantly affected (downregulated) in this pathway by ND-NH_2_ are shown in Figure 9. The canonical pathway analysis also revealed that the “dendritic cell maturation” pathway was affected in THP-1 cells albeit not significantly for all three NDs (Figure 8B).

**Figure 8:**
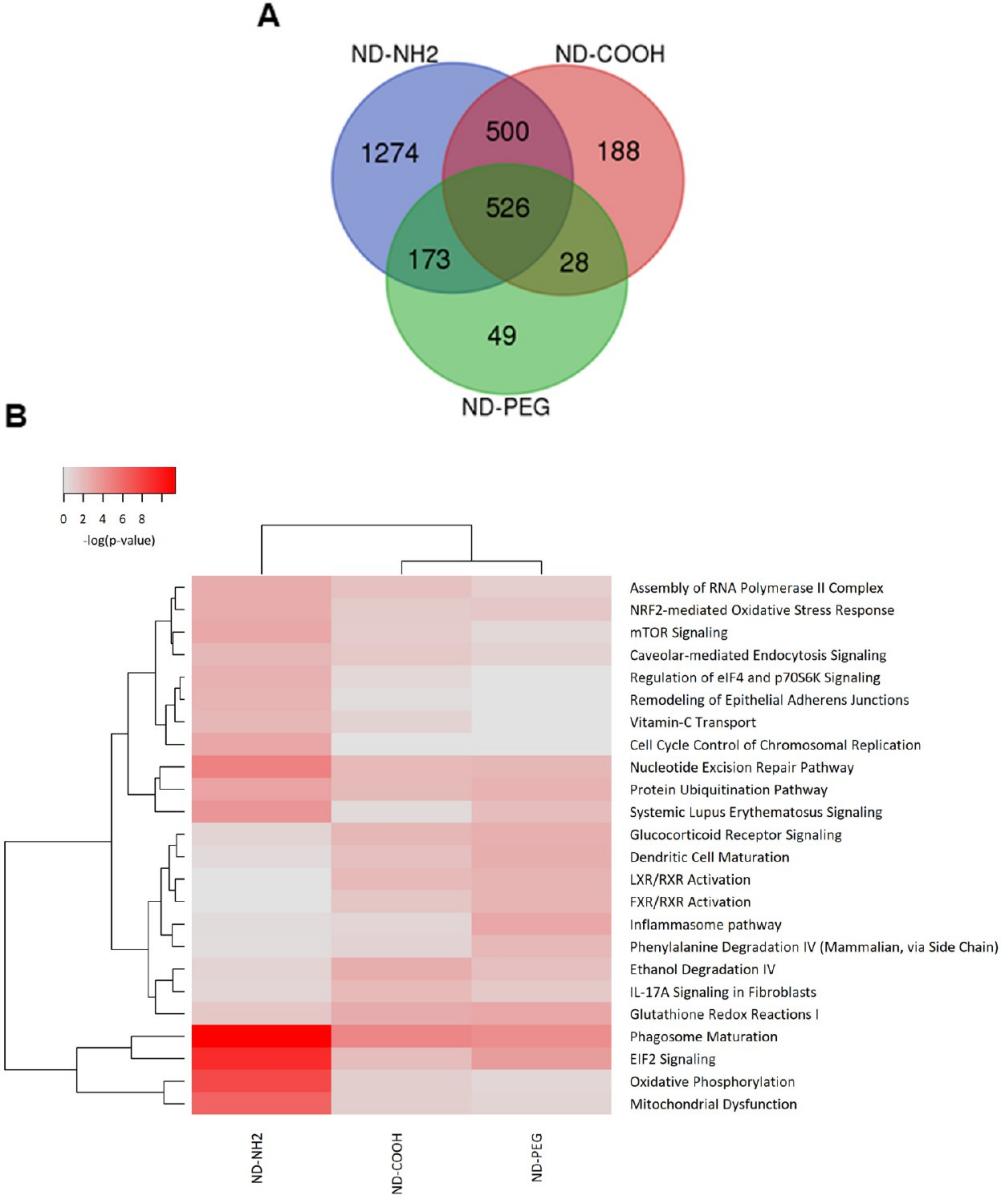
NDs affect phagosome maturation. (A) Venn diagrams of differentially expressed genes (DEGs) as determined by RNA sequencing of THP-1 cells exposed to NDs for 24 h. DEGs having >0.5 log fold change and <0.05 FDR were included in the analysis. (B) Pathway analysis of significant DEGs regulated in THP-1 cells following exposure to NDs (20 μg/mL) for 24 h. The canonical pathway analysis was performed by means of the Ingenuity Pathway Analysis (IPA) software (Qiagen). The significance values for the canonical pathways were calculated by Fisher’s exact test right tailed, and indicates the probability of association of the DEGs with the respective pathway. The heatmap was generated using canonical pathways filtered on *p* < 0.01.

**Figure 9:**
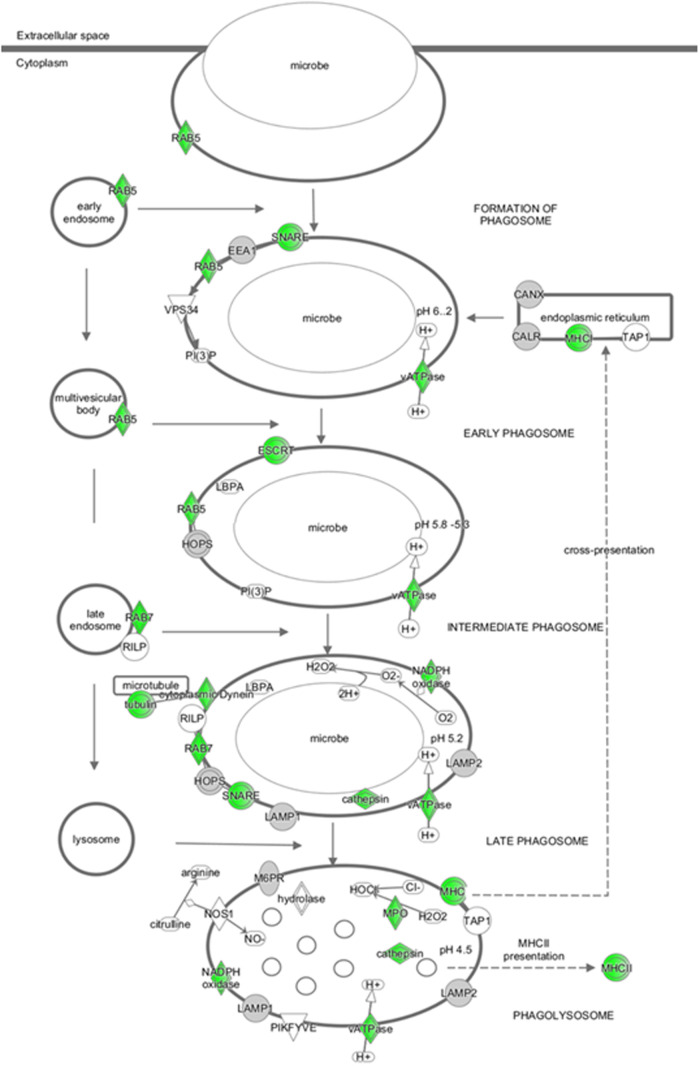
NDs affect genes involved in phagosome maturation. IPA analysis of RNA-sequencing data retrieved from THP-1 cells exposed to ND-NH_2_ (20 μg/mL) for 24 h revealed perturbations in the “phagosome maturation” pathway, with significant downregulation (green) of several key genes. The diagram was generated using the Ingenuity Pathway Analysis (IPA) software (Qiagen). The term ‘microbe’ was introduced by the IPA software; however, in this case, ‘microbe’ may be replaced with NDs.

### Evidence for ND-Triggered Lysosomal Stress

The transcriptomics study using THP-1 cells thus implicated phagosomes or their maturation, while our investigations of primary human DCs (MDDCs) and macrophages (HMDMs) (and THP-1 cells) suggested that NDs are trafficked to the endosomal-lysosomal compartment. To further investigate the cellular fate of the NDs, and to explore whether phagosome maturation was affected, MDDCs exposed to NDs (25 μg/mL) for 24 h were labeled with LysoTracker and LysoSensor, two fluorescent dyes that stain acidic compartments in the cell, such as lysosomes. We found that ND-NH_2_, ND-COOH, and ND-PEG all triggered intense punctate staining with LysoTracker (red) while LysoSensor staining (green) was also observed, though this was less pronounced (Figure S12A). The same experiment was performed using HMDMs, and we observed intense punctate staining with LysoTracker in cells exposed to ND-NH_2_ (Figure 10A). This was sensitive to treatment with bafilomycin A1 (Figure 10B), confirming that the acidification was dependent on the vacuolar H^+^-ATPase.^41^ Similar results were obtained for ND-COOH and ND-PEG (data not shown).

**Figure 10:**
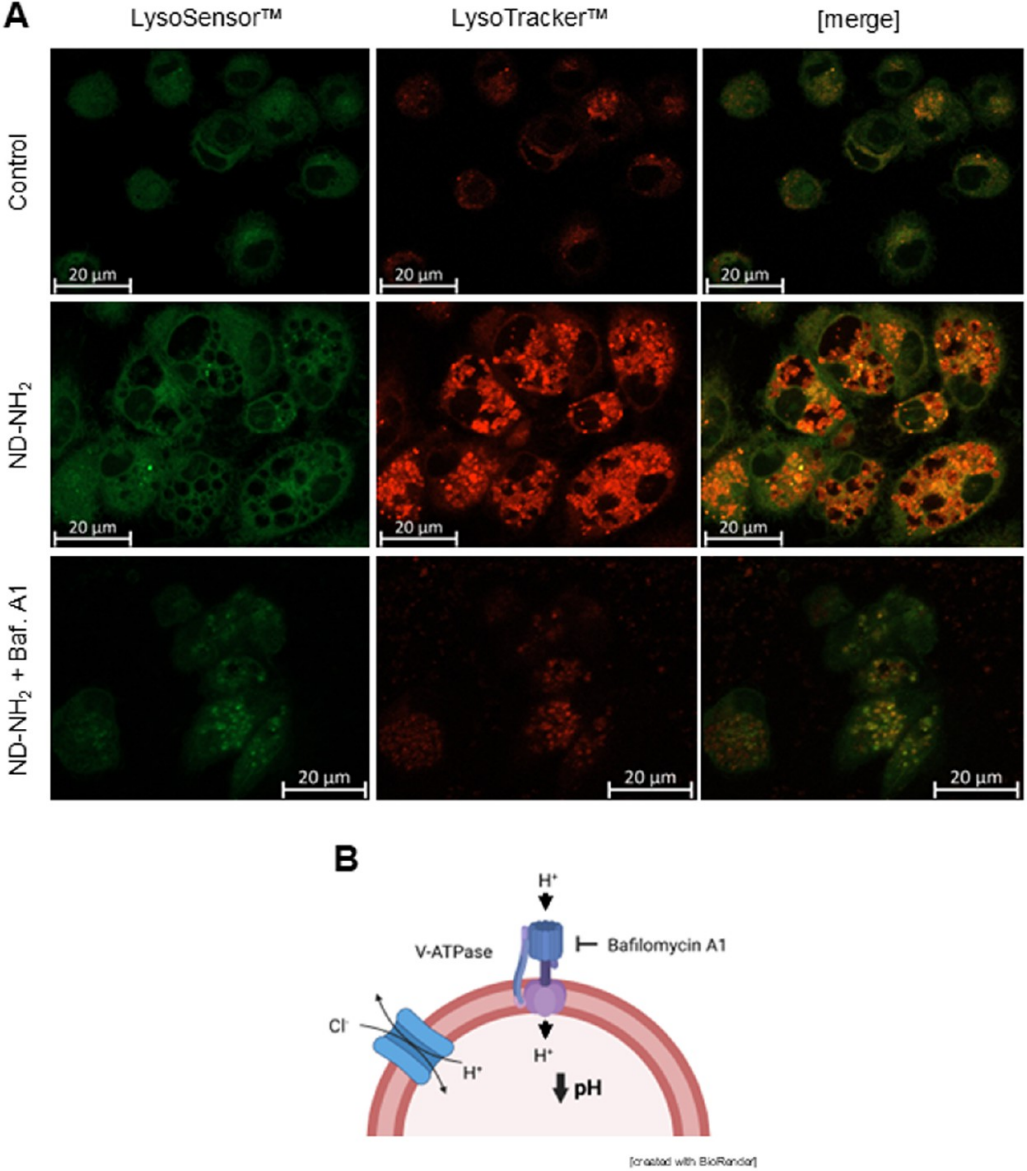
NDs trigger hyperacidification of lysosomes. (A) HMDMs were exposed for 24 h to ND-NH_2_ (25 μg/mL) with or without bafilomycin A1 (10 nM) and stained with LysoTracker (red) and LysoSensor (green). The DAPI filter was removed for better visualization. Samples were imaged by confocal microscopy. Scale bars: 20 μm. Refer to Figure S12A for corresponding results for MDDCs exposed to NDs. (B) Schematic rendition of the vacuolar-type H^+^-ATPase (V-ATPase) and its inhibition by bafilomycin A1. (B) was generated by using BioRender.com under an academic license.

Lysosomes are degradative organelles, and their luminal pH is normally maintained at pH 4.5–5.0.^42^ The present results are indicative of the hyperacidification of lysosomes. The lysosome-associated membrane proteins LAMP-1 and LAMP-2 constitute more than 50% of the total membrane protein of late endosomes and lysosomes, and it is presumed that these heavily glycosylated proteins protect lysosomal membranes from luminal hydrolases.^43^ However, LAMP proteins are not merely structural components, and recent work implicated LAMP-1 and LAMP-2 in the regulation of lysosomal pH through direct binding and inhibition of a cation channel.^44^ We decided to monitor the expression of LAMP-1 in cells exposed to NDs. To this end, HMDMs were exposed to ND-NH_2_ (25 μg/mL), and cells were stained with specific antibodies against LAMP-1. We observed a strong upregulation of LAMP-1 expression in ND-exposed cells when compared to untreated control cells (Figure 11A). The upregulation of LAMP-1 expression thus correlated with the lysosomal hyperacidification (see above), and could be perceived as a cytoprotective response to the NDs. Most acidic hydrolases do not function effectively in either hypo-acidified or hyper-acidified lysosomes. To provide further evidence for lysosomal stress, we investigated the processing of cathepsin B (a lysosomal cysteine protease) in HMDMs exposed to NDs. Cathepsin B is synthesized as an inactive zymogen and transferred to endolysosomes where inactive cathepsin B is processed into its mature form. We observed that control cells expressed the heavy chain of the mature double-chain isoform (25/26 kDa) while cells exposed to ND-NH_2_, ND-COOH, or ND-PEG displayed impaired maturation of cathepsin B, as evidenced by the expression of the mature single-chain (31 kDa) isoform as well as the heavy chain of the mature double-chain isoform (Figure 11B). Treatment of cells with bafilomycin A1 completely prevented processing of cathepsin B. We noted a similar impairment of cathepsin B maturation in MDDCs (Figure S12B).

**Figure 11:**
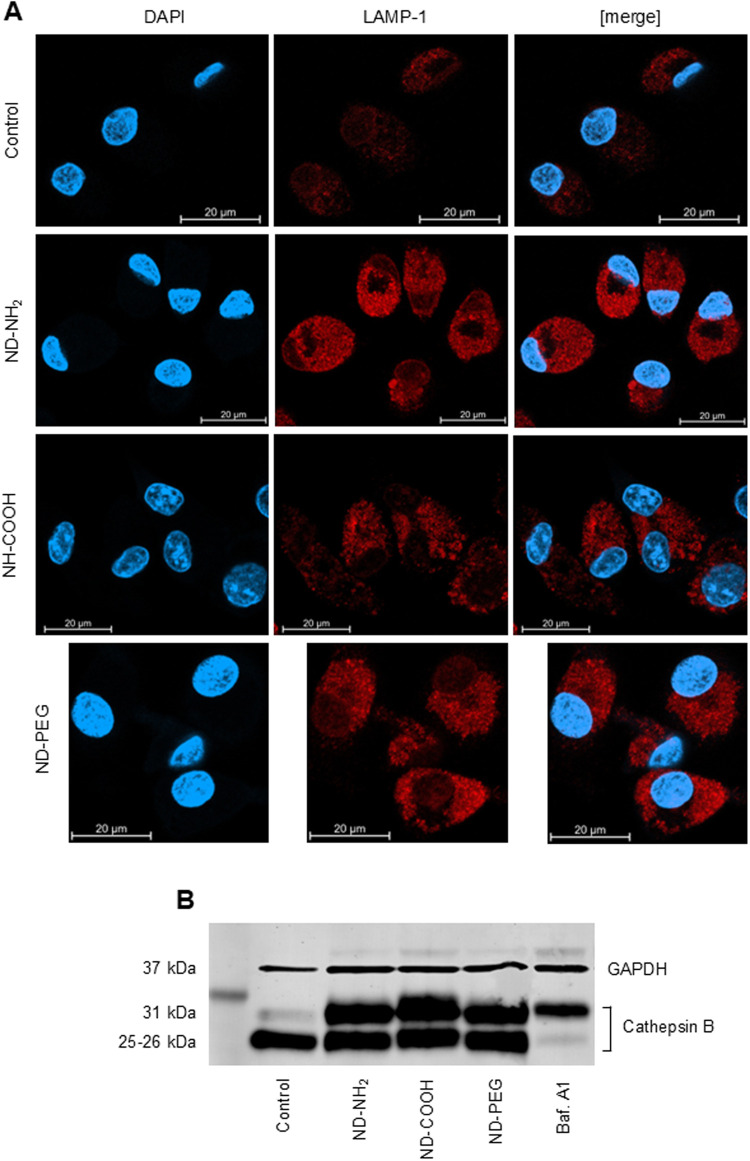
NDs elicit signs of lysosomal stress. (A) HMDMs were exposed for 24 h to ND-NH_2_, ND-COOH, or ND-PEG (25 μg/mL) versus control. Cells were stained with antibodies against lysosomal-associated membrane protein-1 (LAMP-1) (and Alexa Fluor 594-conjugated secondary antibody) (red) and counterstained with DAPI (blue). Samples were imaged by confocal microscopy. Scale bars: 20 μm. (B) HMDMs were exposed for 24 h to NDs with different surface functionalities (25 μg/mL). Bafilomycin A1 (10 nM) was applied to inhibit the vacuolar H^+^-ATPase. Cathepsin B expression and processing was monitored by Western blot. GAPDH was used as a loading control. Refer to Figure S12B for corresponding Western blot results for MDDCs exposed to NDs.

Lysosomal hyperacidification is considered a hallmark of autophagy.^45^ To test whether NDs triggered autophagy in immune cells, we deployed the murine RAW-Difluo mLC3 reporter cell line. These cells are engineered to express LC3B (microtubule-associated protein 1 light chain 3 β) fused to two fluorescent reporter proteins, RFP (acid-stable) and GFP (acid-sensitive), thus allowing for the measurement of autophagic flux.^46^ Hence, the tandem fluorescent-tagged LC3 makes it possible to distinguish autophagosomes (GFP- and RFP-positive puncta, which are yellow) from acidic autolysosomes (GFP-negative and RFP-positive puncta, which are red). We observed predominantly yellow LC3 puncta upon ND exposure (Figure S13), indicating the presence of autophagosomes. TEM analysis of RAW264.7 (nonreporter) macrophages exposed to NDs confirmed the presence of autophagosomes (Figure S14). Notably, autophagosome accumulation may result from blockade of autophagy flux rather than autophagy induction.^47^ Indeed, NDs were previously suggested to act as autophagy inhibitors, thereby augmenting cancer therapy.^48,49^ For comparison, we studied control nanoparticles (TiO_2_–NH_2_, TiO_2_–COOH, and TiO_2_–PEG), and observed that these particles mainly provoked red puncta in the macrophage reporter cell line, suggestive of autolysosomes (Figure S15). Overall, our data demonstrated that while NDs are biocompatible with respect to immune cells, signs of lysosomal stress are noted. Lysosomes generate and maintain their pH gradient through the proton pumping activity of the vacuolar H^+^-ATPase, and this acidification requires the parallel movement of neutralizing counterions.^50^ It is notable that NDs have been shown to act as a Trojan horse for monovalent and divalent ions.^51,52^ From a chemobiological point of view, it may therefore be of interest to study whether NDs promote lysosomal acidification by directly influencing the passage of protons and/or counterions. From a conceptual standpoint, it may be instructive to consider NDs as persistent (nondegradable) “pathogens” provoking lysosomal hyperacidification in phagocytic cells.^53^

### NDs Trigger TLR-Dependent IFN-α Release

Primary human macrophages and DCs are commonly generated *ex vivo* through the differentiation of monocytes present in peripheral blood. We have shown here, using HMDMs and MDDCs, that ND-NH_2_, ND-COOH, and ND-PEG are taken up by cells and trafficked to the lysosomal compartment; we also observed signs of lysosomal stress albeit in the absence of cytotoxicity at concentrations up to 100 μg/mL. We also used monocyte-like THP-1 cells and macrophage-like RAW264.7 cells to investigate the effects of NDs on immune-competent cells. However, our initial findings using PBMCs (see above) demonstrated that NDs increased the number of pDCs, a rare cell type whose function may not be accurately recapitulated by *ex vivo* generated DCs.^54^ Therefore, we turned again to human PBMCs, and asked whether the exposure to NDs could trigger the release of the type I interferon, IFN-α. Exposure of PBMCs to CpG-A (a TLR9 ligand consisting of synthetic oligonucleotides) triggered robust IFN-α production, thus validating PBMCs as a model (Figure S16A). Moreover, IFN-α production was suppressed by cytochalasin D, as well as by the MyD88 inhibitor, Pepinh-MYD, and the NFκB inhibitor, Bay 11–7082, thus confirming the canonical pathway of IFN-α production (Figure S16A). Previous work has shown that autophagy is required for the production of IFN-α in pDCs.^55^ We therefore tested whether wortmannin, a selective inhibitor of phosphoinositide 3-kinase (PI3K) activity which is essential for the induction of autophagy,^56^ or chloroquine, a compound that inhibits autophagy by impairing autophagosome fusion with lysosomes,^57^ affected IFN-α production. Both inhibitors blocked the IFN-α release caused by CpG-A (Figure S16B), confirming once again that PBMCs are a valid model. We then exposed PBMCs for 24 h to ND-COOH, ND-NH_2_, or ND-PEG (50 μg/mL). We opted for the latter concentration as our pilot studies indicated that 25 μg/mL did not yield a robust IFN-α response in PBMCs (data not shown). We detected a response to all three NDs albeit lower than the positive control, CpG-A (Figure 12A). IFN-α production was blocked by cytochalasin D (Figure 12A), implying that cellular uptake of NDs was required, and we confirmed that the MyD88-NFκB-dependent pathway was involved, as shown in Figure 12B. These results thus point to the role of the classical TLR-dependent induction of IFN-α. We also addressed whether the TANK-binding kinase 1 (TBK1), involved in the alternative nonendosomal pathway of interferon production,^58^ was involved by preincubating cells with MRT6737, an inhibitor of IKKε and TBK1. However, this only marginally decreased the IFN-α production caused by ND-NH_2_ and failed to block IFN-α production in cells exposed to ND-COOH and ND-PEG (Figure 12C). We then asked whether autophagy inhibitors prevented IFN-α production in ND-exposed cells. Indeed, wortmannin and chloroquine suppressed IFN-α release in response to NDs (Figure 12D). In a previous study, gene expression profiling using polymerase chain reaction arrays revealed that *IFNA1* was the most upregulated gene in PBMCs exposed to surface modified NDs.^23^ However, the potential role of DCs was not disclosed. Here, we revealed for the first time that NDs triggered IFN-α release, pointing to the involvement of pDCs, and we found that this was autophagy-dependent.

**Figure 12:**
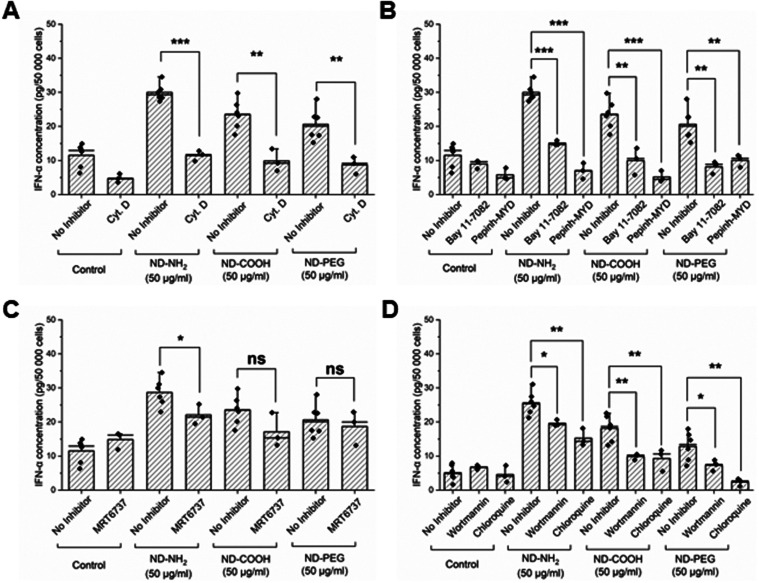
NDs trigger IFN-α release. IFN-α release was evaluated in PBMCs exposed for 24 h to NDs (50 μg/mL) in the presence or absence of the endocytosis inhibitor cytochalasin D (A), the MyD88 inhibitor Pepinh-MYD, or NFκB inhibitor Bay 11–7082 (B), the TBK1 inhibitor MRT6737 (C), or the PI3K inhibitor wortmannin and autophagy inhibitor chloroquine (D). Student’s *t* test was applied. *p ≤ 0.05, **p ≤ 0.01, ***p ≤ 0.001. Refer to Figure S16 for results using the positive control, i.e., TLR agonist, CpG-A.

### NDs Activate Endosomal TLR7 and TLR9

Unlike other DCs, pDCs do not express TLR2 or TLR4 which explains why these cells do not respond to bacterial products such as lipopolysaccharide (LPS).^33^ Instead, pDCs are highly specialized in the defense against viruses, and viruses trigger IFN responses in pDCs through endosomal TLR7 and TLR9 receptors which signal through MyD88, which, in turn, activates the transcription factor NF-κB. Type I IFNs secreted by pDCs promote natural killer (NK) cell and cytotoxic T cell activity to kill virus-infected cells.^33^ To address the potential role of endosomal TLRs for the intracellular “sensing” of NDs, we used reporter cell lines engineered to express human TLR7 (HEK-Blue hTLR7) or human TLR9 (HEK-Blue hTLR9). We applied imiquimod (TLR7 agonist) and CpG-A (TLR9 agonist) as positive controls (Figure 13A,B). Both ND-NH_2_ and ND-COOH elicited a dose-dependent activation of TLR7, while the effect of ND-PEG was not statistically significant (Figure 13C). Concerning TLR9, all three NDs triggered a dose-dependent activation, but the response to ND-PEG was lower than for the other NDs (Figure 13D). Overall, the experiments conducted in these human reporter cell lines have shown for the first time that NDs are capable of triggering endosomal TLRs (specifically, TRL7 and TLR9), although the response was less pronounced for ND-PEG in this model. This provides a plausible mechanism for the induction of IFN-α production observed in PBMCs. Recent work has shown that calcium nanoparticles can be used as a vehicle for the delivery of calcium ions thereby modulating DC function, likely by triggering NF-κB and NFAT signaling pathways.^59^ However, the present study is the first to suggest that engineered nanomaterials trigger DCs through the engagement of endosomal receptors. NDs could, potentially, interact indirectly with TLR7/TLR9 by virtue of a “corona” of nucleic acids, which are the natural ligands for these receptors. Interestingly, other investigators demonstrated that polystyrene (PS) nanoparticles mixed with DNA could trigger cytokine responses in RAW264.7 macrophages.^60^ The authors found that the cyclic GMP-AMP synthase (cGAS)-stimulator of interferon genes (STING) pathway was responsible for the “sensing” of the DNA-PS complexes. Thus, if NDs were to encounter and interact with (nonself) DNA, this might explain the subsequent activation of cellular receptors. However, previous computational studies suggested that the internal hydrophobic pockets of some TLRs might be capable of directly binding carbon-based nanostructures, e.g., fullerenes.^61^ Moreover, our combined experimental and theoretical results revealed that single-walled carbon nanotubes (CNTs) could bind to TLR2 and TLR4 on the cell surface.^62^ We therefore examined the binding of individual functionalized NDs to endosomal TLR7 and TLR9 by molecular docking experiments. Imiquimod, a synthetic, nucleotide-like TLR agonist, was included as a reference. For these computational studies, the docking grid was set to encompass the ligand-binding domain (LBD) of both receptors. We found that NDs interacted with high binding affinities with the ring-like structures of the LBDs. Regardless of surface functionalization, NDs were docked into the same position within the LBDs indicated by low values or upper bound root-mean-square deviation (of atomic positions) (RMSD) of individual binding poses while imiquimod exhibited different binding poses on the surface of the LBDs (Figure S17A,B). The greatest binding affinities were observed for ND-NH_2_ followed by ND-COOH whereas binding affinities for ND-PEG were lower for TLR7 and TLR9 (Figure S17A,B). Taken together, we favor the view that the NDs bind directly to endosomal TLRs.

**Figure 13:**
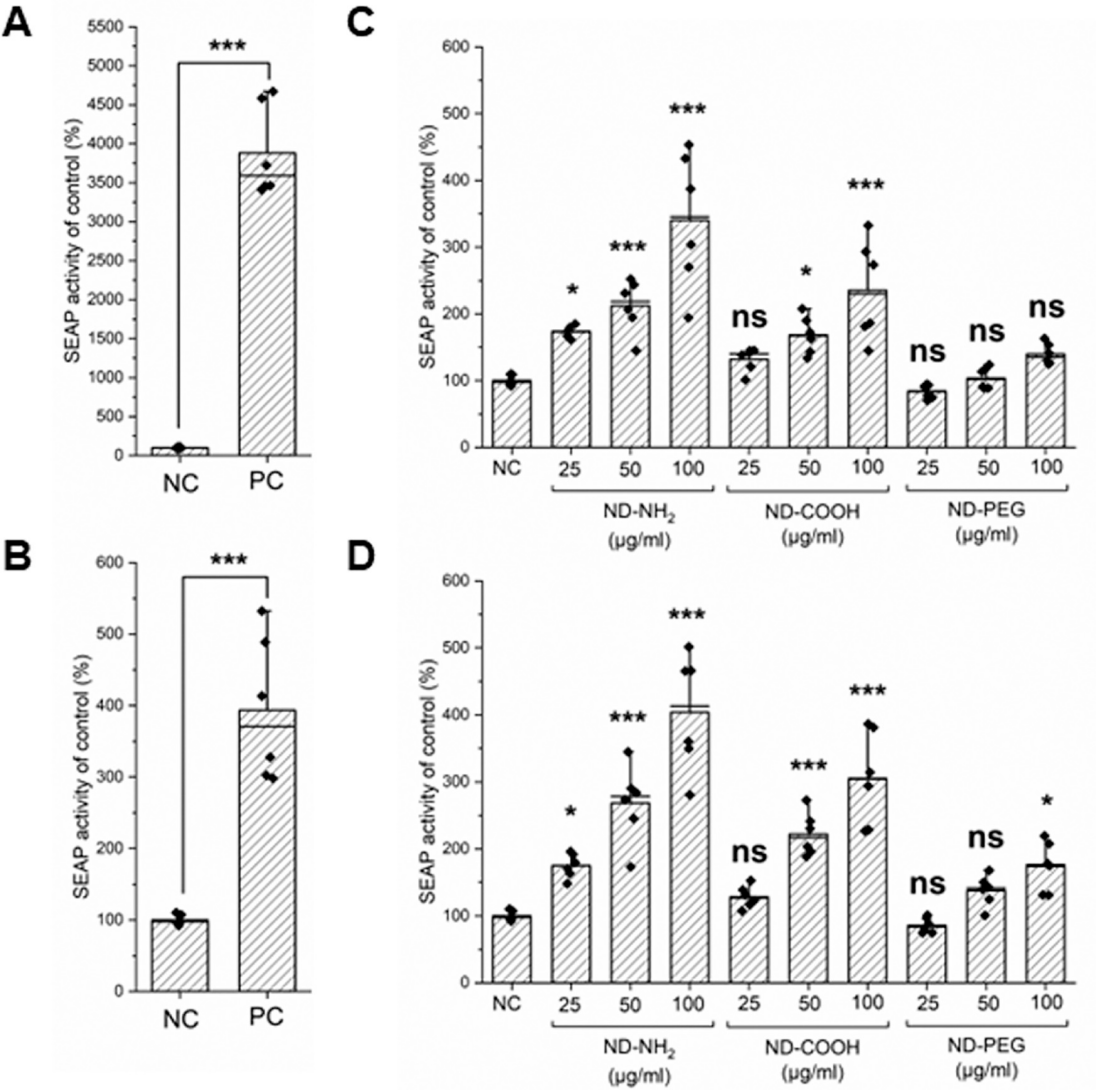
NDs trigger activation of TLR7 and TLR9. SEAP production in (A) HEK-Blue hTLR7 and (B) HEK-Blue hTLR9 reporter cells exposed to imiquimod and CpG-A. The HEK-Blue hTLR7 (C) and HEK-Blue hTLR9 (D) were exposed to the indicated concentrations of ND-NH_2_, ND-COOH, and ND-PEG for 24 h. Data were analyzed using one-way Anova with Dunnett’s post hoc test. *p ≤ 0.05, **p ≤ 0.01, ***p ≤ 0.001.

Finally, to ascertain that the observed effects were specific, we tested control nanoparticles with similar surface functionalities (TiO_2_–NH_2_, TiO_2_–COOH, and TiO_2_–PEG, along with nonfunctionalized particles). The hydrodynamic diameters of these particles were comparable to those of the NDs (on average around 150 nm), and the ζ-potential in cell culture medium supplemented with serum was around −10 mV for all the particles (data not shown). We previously demonstrated that these TiO_2_ particles were noncytotoxic for nondifferentiated THP-1 cells and HMDMs.^16^ Here, we tested whether TiO_2_ nanoparticles triggered IFN-α production in PBMCs. To this end, PBMCs from normal donors were exposed to TiO_2_–NH_2_, TiO_2_–COOH, and TiO_2_–PEG, and nonfunctionalized TiO_2_ (50 ug/mL) for 24 h. CpG-A was applied as a positive control. TiO_2_ nanoparticles failed to evoke IFN-α (Figure S18). Furthermore, we applied reporter cell lines to evaluate the activation of TLR7 (HEK-Blue hTLR7) and TLR9 (HEK-Blue hTLR9). No activation of TLR7 or TLR9 was detected when the cells were exposed to TiO_2_ (Figure S19A,B). Thus, we confirmed that the observed effects were specific for NDs.

## Conclusions

We have provided evidence herein that NDs can be “sensed” by primary human immune-competent cells leading to a type I IFN response (Figure 14). Several lines of evidence led us to this conclusion. First, single-cell profiling of PBMCs exposed to NDs revealed an increase in the number of pDCs, a unique subset of DCs specialized in the production of type I IFNs.^33^ Moreover, IFN-α release was observed following the exposure to NDs and this response was dependent on MyD88 and NF-κB indicating a canonical TLR-dependent mechanism of IFN production.^63^ Furthermore, we could show that NDs activate endosomal TLR7 and TLR9, and computational (molecular docking) studies supported the notion that NDs can directly bind TLR7/TLR9. We found that NDs triggered the activation and maturation of human DCs, and our studies have shown that NDs are taken up by MDDCs and HMDMs as well as by HLA-DR-positive antigen-presenting cells or APCs, when incubated with PBMCs, without eliciting cytotoxic responses. Moreover, our transcriptomics data implied an impairment of phagosome maturation, and subsequent experiments revealed lysosomal hyperacidification and impaired maturation of lysosomal hydrolases in macrophages and DCs. Hence, these results imply that NDs are sensed as intracellular “parasites” evoking an innate immune response reminiscent of the immune responses triggered by viruses. In fact, the present study has disclosed that NDs have a dual impact on DCs: the expansion of pDCs as well as the induction of a type I IFN response in pDCs.

**Figure 14:**
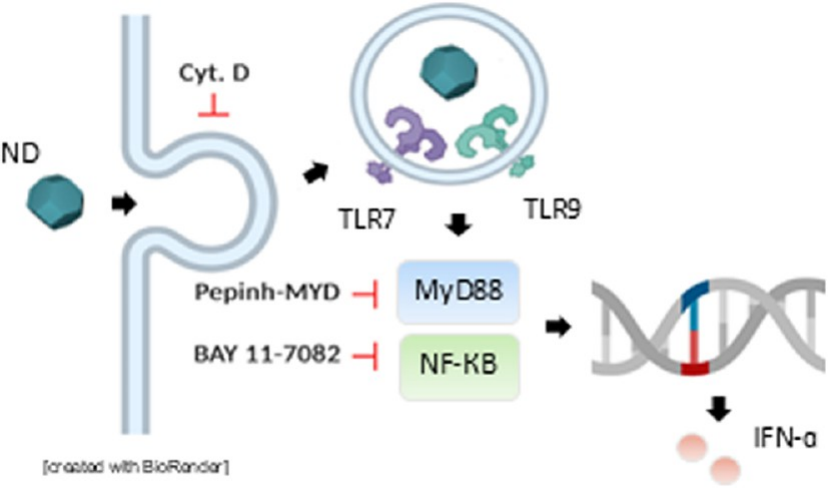
Understanding the intrinsic biological activity of nanodiamonds. The present study has provided evidence that NDs are actively internalized by antigen-presenting cells and trafficked to the endosomal-lysosomal compartment where endosomal Toll-like receptors (TLR7/TLR9) are engaged leading to a type I interferon response. NDs do not compromise cell viability although signs of lysosomal stress were noted (not shown). Figure was generated by using BioRender.com under an academic license.

Notably, while NDs are generally considered biologically “inert”, recent studies have shown that NDs act as autophagy inhibitors in various cancer cells.^48,49^ We could show in the present study that NDs modulated autophagic flux in immune cells, a further sign of lysosomal stress, and we also found that autophagy played a role in IFN-α release, in line with previous work which has disclosed an association between autophagy and antiviral responses.^55^ Furthermore, autophagy, and the related process of LC3-associated phagocytosis (LAP), which represents a convergence of the phagocytic and autophagic pathways,^64^ is known to play a role in antigen presentation. Additionally, maturation of DCs is accompanied by the activation of the vacuolar H^+^-ATPase thereby enhancing lysosomal acidification and antigen proteolysis,^65^ which is linked to antigen presentation.^66^ Further studies are warranted to understand whether antigen presentation by DCs (or macrophages) is affected by NDs.

The NDs that were studied here displayed different surface functionalities (ND-NH_2_, ND-COOH, and ND-PEG), but the average hydrodynamic diameter and ζ-potential were “equalized” when the NDs were immersed in cell culture medium supplemented with 10% FBS, the standard medium used throughout the present study. It is important to recognize that immune cells are capable of decoding the size of particles. Rettig et al. could show, in a very instructive study, that single-stranded RNA (ssRNA) mixed with protamine forms particles and activates immune cells through TLRs in a size-dependent manner.^67^ Hence, using human PBMCs, the authors applied protamine-RNA particles of 220, 500, and 1200 nm, and found that the smaller particles triggered production of IFN-α while the larger particles triggered TNF-α production. This could be explained by the fact that nanoparticles but not microparticles were selectively phagocytosed by pDCs, which produce IFN-α whereas monocytes were found to take up nano- and microparticles similarly, but the threshold of activation of monocytes was higher than the threshold of activation of pDCs. In other words, nanosized particles triggered an antiviral response while micron-sized particles formulated from the same “danger” signal triggered an antibacterial/antifungal immune response.^67^ It is notable that the NDs in the present study formed agglomerates with a diameter of about 150 nm in cell culture medium which might explain the preferential uptake by HLA-DR-positive cells when incubated with total human PBMCs. However, while a similar degree of uptake was noted for all NDs, ND-PEG was less effective in driving IFN-α responses in primary immune cells and reporter cell lines, possibly due to steric hindrance. Moreover, our computational studies indicated that the binding affinity for TLR7/TLR9 was lower for ND-PEG as compared to ND-NH_2_ and ND-COOH. Thus, surface properties, in addition to the size (of the agglomerates), also play a role.

To what extent can the present results inform the clinical use of NDs? First, using primary human immune-competent cells, we confirmed that NDs are biocompatible. Moreover, our results suggest that NDs may be harnessed to modulate DCs. In particular, this would be useful in the context of anticancer vaccines where immune responses with antiviral-like features may be desirable in order to provoke robust responses against tumors.^68,69^ Considerable efforts have been devoted to achieving passive or active targeting of tumor cells.^70^ However, nanomaterial targeting of immune cells to facilitate antitumor responses could serve as a complementary approach.^71,72^ Thus, drug-free NDs (i.e., without any specific therapeutic cargo) could potentially be applied to evoke antitumor immune responses. Moreover, NDs have been applied successfully as drug delivery vehicles in preclinical studies.^73^ Overall, NDs emerge as versatile and biocompatible nanomaterials for biomedical applications.

## Experimental Section

### ND Characterization

NDs with three different surface functionalities (ND-NH_2_, ND-COOH, and ND-PEG) were provided by PlasmaChem GmbH (Berlin, Germany). For details regarding the synthesis and functionalization, refer to Gallud et al.^16^ Nonfunctionalized and functionalized TiO_2_ nanoparticles were also provided by PlasmaChem GmbH. The size and morphology of the NDs was investigated using the Hitachi HT 7700 electron microscope (Hitachi High-Technologies). High-resolution (HR) TEM (EM-2100 F, 200 kV, JEOL) was applied to identify the diamond crystalline plane in ND-COOH. The hydrodynamic diameter and ζ-potential of NDs dispersed in Milli-Q water and RPMI-1640 medium supplemented with 10% FBS, and of TiO_2_ nanoparticles in DMEM supplemented with 10% FBS, was determined by using the Malvern Zetasizer Nano ZS.

X-ray photoelectron spectroscopy (XPS) and Fourier transform infrared spectroscopy (FTIR) were used to analyze the surface chemistry of the three ND samples. FTIR was performed using an iS5 Thermo Nicolet spectrometer (with ZnSe ATR technique). XPS was performed as described previously.^74^ The measurements were carried out using the Nexsa G2 XPS system (Thermo Fisher Scientific) with monochromatic Al Kα source and photon energy of 1486.7 eV. The spectra were measured in the vacuum of 1.2 × 10^–7^ Pa and at the room temperature of 20 °C. The analyzed area of each sample was a spot with a radius of 200 μm. The survey spectra were measured with a pass energy of 150.00 eV and an electronvolt step of 1.0 eV while for the high-resolution spectra, a pass energy of 30.00 eV and an electronvolt step of 0.1 eV were used. Charge compensation was applied for all measurements. The spectra were evaluated and plotted with Avantage version 6.5.1 software (Thermo Fisher Scientific).

### Endotoxin Assessment

To detect potential endotoxin contamination, we used the Endpoint Chromogenic LAL assay (Lonza) as previously described.^19^ NDs alone, NDs spiked with LPS, and NDs spiked with LPS in the presence of polymyxin B sulfate (Sigma-Aldrich) were tested. Endotoxin results for the TiO_2_ nanoparticles used in this study were previously reported; refer to Gallud et al.^16^

### Primary Immune Cells

Several populations of primary human immune cells were used in this study. First, PBMCs were isolated from buffy coats obtained from adult human blood donors (Karolinska University Hospital, Stockholm) using gradient centrifugation with Lymphoprep (Stemcell Technologies).^75^ The identity of the blood donors was unknown to the researchers, and data cannot be traced back to the individual donors. Therefore, a specific permit was not required. Experiments were carried out in accordance with KI guidelines for the handling of blood (1–505/2022). CD14-positive monocytes were isolated using CD14 MicroBeads (Miltenyi Biotec Ltd.). The monocytes were then differentiated into either HMDMs or MDDCs. The cells were cultivated at 37 °C in a humidified atmosphere in a 5% CO_2_ incubator in RPMI-1640 medium (Gibco) supplemented with 10% heat-inactivated FBS (Gibco), 1% l-glutamine (Sigma-Aldrich), and 1% penicillin/streptomycin (Gibco). To generate HMDMs, monocytes were maintained for 4 days in RPMI-1640 medium supplemented with 50 ng/mL recombinant M-CSF (PeproTech). Additionally, to generate MDDCs, monocytes were incubated with 80 ng/mL of GM-CSF (PeproTech) and 50 ng/mL of IL-4 (Sigma-Aldrich) supplemented complete RPMI-1640 medium for 48 h. Then, half of the medium was replaced with fresh medium containing the same amounts of cytokines and the cells were incubated for a further 72 h. On day 5, MDDCs displayed a phenotype of immature DCs, and were subsequently used for experiments.

### Human and Murine Cell Lines

The murine macrophage-like cell line RAW264.7 from the European Collection of Cell Cultures (ECACC) was cultured in Dulbecco’s modified Eagle’s medium (DMEM) supplemented with 10% heat-inactivated FBS (Gibco), 1 mM sodium pyruvate, and 1% penicillin/streptomycin (Gibco). Cells were seeded in 96-well plates (3.5 × 10^4^ cells/well) and incubated for 24 h at 37 °C and 5% CO_2_ prior to experiments. Thereafter, cells were exposed to the indicated concentrations of NDs for 24 h, and monitored with respect to uptake of NDs. The human acute monocytic leukemia cell line THP-1 was purchased from the American Type Culture Collection (ATCC). Cells were maintained in RPMI-1640 medium supplemented with penicillin/streptomycin and 10% heat-inactivated FBS. The cells were applied for transcriptomics and TEM, as described below. Both cell lines were tested regularly using MycoAlert mycoplasma kit (Lonza).

### Single-Cell Mass Cytometry

To perform single-cell mass cytometry, we followed a previously described protocol.^31^ PBMCs were obtained from healthy adult donors as described above. PBMCs were seeded at a concentration of 4.0 × 10^6^ cells per well in 6-well plates. The cells were then exposed to ND-COOH, ND-NH_2_, or ND-PEG at a concentration of 20 μg/mL for 24 h. LPS (Sigma-Aldrich) at a concentration of 0.1 μg/mL was used as a positive control, and untreated cells served as the negative control. After exposure, the cells were stained with Cell-ID Intercalator-103Rh (Fluidigm, CA) at a dilution of 1:500 for 15 min at 37 °C. Subsequently, they were washed and combined using the Cell-ID 20-Plex Pd Barcoding Kit (Fluidigm). The Maxpar Human Peripheral Blood Phenotyping and Human Intracellular Cytokine I Panel Kits (Fluidigm) were used to stain the cells according to the manufacturer’s protocol. Each antibody in the cocktail was used at a dilution of 1:100. Following the incubation, the cells were incubated with Cell-ID Intercalator-Ir solution at a final concentration of 125 nM for 5 min. Cell-ID Intercalator-Ir is a cationic nucleic acid-intercalating molecule used to distinguish live cells from dead cells. To eliminate possible cell clusters or aggregates, each sample was filtered through a 0.22 μm cell strainer cap before data acquisition. The mass cytometry data were analyzed using the CyTOF 2 platform (Fluidigm Corporation, CA). The normalized background subtracted data files were uploaded into Cytobank for analysis. Cell events were gated to exclude doublets, and cell debris. Specific subpopulations were defined as shown (Figure S20). The markers used for defining the subpopulations were as follows: T cells (CD45+ CD19– CD3+), T helper (CD45+ CD3+ CD4+), T cytotoxic (CD45+ CD3+ CD8+), T naive (CD45RA+ CD27+ CD38– HLA-DR−), T effector (CD45RA+ CD27– CD38– HLA-DR−), activated T cells (CD38+ HLA-DR+), B cells (CD45+ CD3– CD19+), B naive (HLA-DR+ CD27−), B memory (HLA-DR+ CD27+), plasma B (HLA-DR– CD38+), NK cells (CD45+ CD3– CD19– CD20– CD14– HLA-DR– CD38+ CD16+), classical monocytes (CD45+ CD3– CD19– CD20– HLA-DR+ CD14+), intermediate monocytes (CD45+ CD3– CD19– CD20– HLA-DR+ CD14dim CD16+), nonclassical monocytes (CD45+ CD3– CD19– CD20– HLA-DR+ CD14– CD16+), myeloid DCs (CD45+ CD3– CD19– CD20– CD14– HLA-DR+ CD11c+ CD123−), plasmacytoid DCs (CD45+ CD3– CD19– CD20– CD14– HLA-DR+ CD11c– CD123+). The resulting data were visualized using viSNE,^76^ a visualization software which employs *t*-stochastic neighbor embedding (*t*-SNE). To generate the map, nine cell surface markers were used: CD3, CD4, CD8a, CD11c, CD14, CD16, CD19, CD20, CD123, and HLA-DR. The viSNE tool was then used to assess viability data analysis. Plots displaying the expression intensity of LD signal and heatmaps of the mean expression ratios were generated to further analyze the data.

### Cell Viability Assays

To assess the potential cytotoxicity of NDs, we applied the LDH release assay and Alamar Blue assay, as previously described.^74^ Briefly, cell supernatants were collected to measure LDH release using the CytoTox 96 assay kit (Promega, Madison, WI) following the manufacturer’s instructions LDH release was normalized to the maximum LDH induced by the cell lysis buffer provided in the kit. For measurements of metabolic capacity, Alamar Blue reagent was prepared freshly in RPMI-1640 cell medium according to the manufacturer’s instruction (ThermoScientific). The exposed cells were rinsed with PBS and Alamar Blue reagent was added to each well. Following incubation for 3 h at 37 °C, fluorescence was measured at the respective excitation and emission wavelength of 531 and 595 nm using a Tecan Infinite F200 plate reader (Tecan, Stockholm, Sweden). The results were normalized to the untreated negative controls.

### Cellular Uptake

To detect the uptake of NDs in PBMCs, we applied a label-free approach.^34^ Briefly, 5 × 10^5^ PBMCs were seeded in 12-well plate and exposed to 25 μg/mL of NDs for 24 h. Then, the samples were collected, washed once with PBS, and analyzed using BD LSRFortessa X-20 (BD Biosciences, San Jose, CA). To investigate the mechanism of uptake of NDs, samples were preincubated for 1 h with or without cytochalasin D (10 μM). Additionally, staining with FITC Mouse Anti-Human HLA-DR (BD Biosciences) was performed to evaluate the uptake of NDs in HLA-DR-positive cells (using PBMCs).

### Confocal Microscopy

Identification (localization) of lysosomes in HMDMs and MDDCs was assessed by confocal imaging. To this end, CD14-positive monocytes (5 × 10^5^) were seeded and differentiated in μ-Slide 18-well IbiTreat chamber (Ibidi). After differentiation, cells were exposed for 24 h to NDs (25 μg/mL) with/without bafilomycin A1 (Sigma-Aldrich) to block lysosomal acidification, and lysosomes were visualized using two different protocols. First, LysoTracker Red (Thermo Fisher Scientific) or LysoSensor Green (Invitrogen) was used. Following treatment, culture medium was discarded, cells were washed once with warm PBS, fresh culture medium with LysoTracker Red (50 nM) or LysoSensor Green (1 μM) was added and cells were incubated for 1 h in the cell incubator. Then, the medium was discarded, cells were washed once with warm PBS, fresh culture medium was added, and cells were imaged using a Zeiss LSM900-Airy confocal microscope (Zeiss). Alternatively, immunostaining with mouse antibody against the lysosomal transmembrane protein LAMP-1 (Abcam) and counterstaining with DAPI (Abcam) was performed. Briefly, following 24 h of incubation with NDs, cells were fixed for 10 min using a 4% paraformaldehyde solution (Sigma-Aldrich), permeabilized for 10 min with PBS + 0.1% Triton-X (Sigma-Aldrich), blocked for 30 min with Odyssey Blocking Buffer (LI-COR Biosciences), stained for 1 h with primary antibody against LAMP-1 (1:100 dilution), stained for 1 h with secondary antibody (Alexa Fluor 594 goat anti-Mouse, Thermo Fisher, 1:1000 dilution), stained with DAPI (5 μM) for 15 min, and imaged using a Zeiss LSM900-Airy confocal microscope.

### TEM and SEM

TEM was performed to visualize cellular uptake of NDs and TiO_2_ nanoparticles.^77^ Briefly, cells were exposed to 25 μg/mL of NDs for 24 h, or 50 μg/mL of TiO_2_ for 6 h, supernatants were discarded, cells were washed with PBS, harvested using trypsin-EDTA (0.25%) and gentle scraping, and finally fixed in 2.5% glutaraldehyde in 0.1 M phosphate buffer, pH 7.4 for 1 h at room temperature. Following postfixation in 1% OsO_4_ in 0.1 sodium phosphate buffer for 1 h at 4 °C, the cells were dehydrated using a gradient of ethanol followed by acetone and LX-112 infiltration and finally embedded in LX-112. Ultrathin sections (50–80 nm) were prepared using a Leica EM UC6 microtome, contrasted with uranyl acetate followed by lead citrate, and examined using a Hitachi HT 7700 electron microscope (Hitachi High-Technologies). The interaction of NDs with MDDCs was evaluated using SEM. The cells were grown and differentiated on plastic coverslips inserted into 6-well plates and then exposed to 25 μg/mL of NDs for 24 h. Then, supernatants were discarded, cells were washed with PBS and fixed using 2.5% glutaraldehyde in 0.1 M phosphate buffer, pH 7.4. Fixed cells adhered onto coverslips were then washed with PBS and Milli-Q water prior to the stepwise ethanol dehydration and critical-point-drying using carbon dioxide (Leica EM CPD300). The coverslips were mounted on alumina specimen pins using carbon adhesive tabs and sputter coated with platinum (Quorum Q150T ES). Images were acquired using an Ultra 55 field emission scanning electron microscope (Zeiss) using the SE2 detector at 3 kV.

### Cell Surface Markers

Cell surface markers were investigated by flow cytometry, as described.^78^ The selected markers (all antibodies conjugated with FITC) were HLA-DR, CD11c, CD123 (BD Biosciences), and CD303 (Miltenyi Biotec) for PBMCs, HLA-DR, CD11c, CD86, CD123 (BD Biosciences) and CD303 (Miltenyi Biotec) for CD14-positive monocytes, and CD86, CD83, CD80, CD40, CD123 (BD Biosciences) and CD303 (Miltenyi Biotec) for MDDCs. As an isotype control, FITC mouse IgG3 (BD Biosciences) was used. Briefly, 2 × 10^5^ cells isolated as described above were seeded in 12-well plates and exposed to 25 μg/mL of NDs for 24 h. The samples were collected (MDDCs were harvested using trypsin-EDTA (0.25%) and gentle scraping), washed once with PBS, stained with specific FITC-conjugated antibodies for 10 min at 4 °C, washed once with PBS, and analyzed using the BD LSRFortessa X-20 (BD Biosciences). Data were analyzed using FCS Express version 7.0 software (DeNovo Software, Pasadena, CA).

### Western Blot Analysis

Cathepsin B expression was monitored by Western blot analysis. Following exposure to the different NDs (25 μg/mL), or to bafilomycin A1, cells were detached using trypsin-EDTA (0.25%) and gentle scraping. Subsequently, the samples were lysed on ice for 1 h using RIPA buffer (50 mM Tris HCl, 150 mM NaCl, 1% Triton X-100, 0.25% sodium deoxycholate, 0.1% SDS, and 1 mM EDTA). The buffer was freshly supplemented with protease and phosphatase inhibitors including Mini EDTA-free Protease Inhibitor Cocktail (Sigma-Aldrich), 1 mM PMSF (Thermo Fisher Scientific), PhosSTOP (Sigma-Aldrich), and 1 mM dithiothreitol (DTT) (Sigma-Aldrich). After centrifugation of the cell lysates at 13,000 rpm for 20 min at 4 °C, the supernatants were collected. The total protein content was determined using the Pierce BCA Protein Assay Kit (Thermo Fisher Scientific), and 30 μg of protein was subsequently loaded onto a NuPAGE 4–12% Bis-Tris gradient gel (Thermo Fisher Scientific). Following electrophoretic separation, proteins were transferred onto a Hybond low-fluorescent 0.2 μm PVDF membrane (Amersham), which was blocked for 1 h in Odyssey Blocking Buffer (LI-COR Biosciences). The primary antibody against cathepsin B (Abcam) was applied and membranes were incubated overnight at 4 °C. GAPDH antibody (Thermo Fisher Scientific) served as the loading control, and the secondary antibody was goat antimouse IRDye 680RD (LI-COR Biosciences). Protein detection was carried out with the LI-COR Odyssey CLx imaging system and Odyssey Image Studio software.

### IFN Production

IFN-α release was determined by ELISA in PBMCs (3 × 10^5^ cells/well in 24-well plates) exposed for 24 h to NDs or TiO_2_ (50 μg/mL). Following incubation, the plates were centrifuged (1500 rpm, 5 min), and supernatants were collected and stored at −80 °C. CpG-A (1 μM) (InVivoGen, Toulouse, France) was used as a positive control. To study the mechanism of IFN-α release, cells were preincubated for 1 h with or without cytochalasin D (10 μM) (Sigma-Aldrich), Pepinh-MYD (5 μM) (InVivoGen), Bay 11–7082 (10 μM) (Selleck Chemicals), the TBK1 inhibitor MRT67307 (10 μM) (InVivoGen), wortmannin (1 μM) (Sigma-Aldrich), or chloroquine (10 μM) (Sigma-Aldrich) and then exposed to NDs or CpG-A for 24 h. IFN-α was detected using IFN α Human ELISA Kit (Invitrogen), and results were calculated as pg/50000 cells using a standard curve.

### Reporter Cell Lines

To investigate the activation of endosomal Toll-like receptors (TLRs), HEK-Blue hTLR7 and HEK-Blue hTLR9 reporter cells (InVivoGen, Toulouse, France) were used. Cells were cultured in DMEM (Gibco) containing 4.5 g/L glucose, 2 mM l-glutamine, 10% FBS, 50 U/mL penicillin, 50 μg/mL streptomycin, and 100 μg/mL of Zeocin (InVivoGen). The activation of TLR7 and TLR9 was assessed by the colorimetric detection of secreted embryonic alkaline phosphatase (SEAP) using HEK-Blue detection medium (InVivoGen). In pilot experiments in which we followed the manufacturer’s protocol, free NDs in the supernatant interfered with the analysis. Therefore, the original protocol was modified to avoid interference. Briefly, 20 μL of NDs or TiO_2_ (final concentration: 25, 50, and 100 μg/mL) or negative control (endotoxin-free water) were added to a 96-well plate, 180 μL of cells were added in DMEM supplemented as above at a density of 80,000 cells/well, and samples were incubated for 6 h. Then, the supernatant was discarded, and wells were washed once with PBS to remove the free NDs. Then, HEK-Blue detection medium was added, and samples were incubated for a further 24 h. Finally, absorbance was measured at 620 nm using the Tecan Infinite F200 plate reader. CpG-A (1 μM) and imiquimod (1 μg/mL) (InVivoGen) were used as positive controls in HEK-Blue hTLR9 and HEK-Blue hTLR7. The autophagy reporter cell line RAW-Difluo mLC3 expressing the RFP:GFP:LC3 fusion protein^79^ was purchased from InVivoGen (Toulouse, France). Cells were maintained in DMEM with 10% FBS, 4.5 g/L glucose, 4 mM l-glutamine, 100 U/mL penicillin, 100 μg/mL streptomycin, and 100 μg/mL Zeocin (InVivoGen). One day prior to each experiment, the cells were seeded on glass coverslips placed in 24 well-plates, and exposed as indicated. Chloroquine (10 μM) (Sigma-Aldrich) was used as a positive control. Then, samples were washed with PBS, fixed with 4% formaldehyde, and counterstained and mounted using Vectashield Antifade Mounting Medium with DAPI (Vector Laboratories, Burlingame, CA). Images were captured with a Zeiss LSM880 confocal microscope equipped with ZEN software.

### RNA Sequencing

THP-1 cells were exposed for 24 h to NDs with different surface modifications at 20 μg/mL followed by RNA extraction. Cells incubated in cell culture medium alone were used as a negative control. After exposure, total RNA was extracted by using the AllPrep DNA/RNA/miRNA Universal Kit (Qiagen). Sequencing libraries were prepared according to a published protocol,^80^ adjusted for 10 ng samples by decreasing the number of cycles to 10 during the first PCR amplification. Sequencing was performed using the Illumina HiSeq2000 sequencing platform. Sequencing results were subjected to quality assessment, alignment, quantitation, and downstream analysis. In brief, the alignment to human genome GRCh37/hg19 was by TopHat2,^81^ quantitation was based on the NCBI RefSeq model, and the differential expression test was SAMstrt.^82^

### Bioinformatics Analysis

Differential gene expression levels between the negative control samples and the NDs (ND-NH_2_, ND-COOH, and ND-PEG) were estimated with a *t*-test and *p*-values were corrected with the Benjamini–Hochberg algorithm (false discovery rate, FDR). Venn diagrams of the DEGs were plotted with the web-based tool, Venny 2.1.0.^16^ Comparative causal network analysis was performed using the Ingenuity Pathway Analysis (IPA) software (QIAGEN Inc., Redwood City, CA) (version 33559992). The significance of the pathways was estimated through the curated Ingenuity knowledge database.^83^ The outputs were filtered by *p*-value <0.005 and activation *Z*-score >2 or < −2. Data were integrated using hierarchical clustering on quantile-normalized data. Values reported in the heatmap are normalized scores of the −log­(*p*-value) with a *p*-value <0.00001 for at least one cluster. Complete linkage and Euclidean distances were employed as metrics to draw association dendrograms between different canonical pathways and the exposure conditions. Statistics were performed using R.^40^

### Molecular Docking

The full-length structures of human TLR7 and TLR9 were obtained from the UniProt database (TLR7, UniProt ID: Q9NYK1; TLR9, UniProt ID: Q9NR96) and predicted using AlphaFold.^84^ For docking experiments, only the monomeric ligand-binding domains (LBDs) were used (residues 31P-830L for TLR7 and 28T-805D for TLR9). The ND structure was obtained from the American Mineralogist Crystal Structure Database^85^ and subsequently modified to a sphere with a diameter of 2 nm. Surface functionalization of NDs was performed using an in-house script, which functionalized 10% of the ND surfaces with amino or carboxy groups, or PEG (one monomer capped with a methyl group). The structure of imiquimod, a TLR7 and TLR9 agonist, was extracted from the crystal structure of the TLR7-imiquimod complex.^86^ Docking experiments involved docking of individual functionalized NDs and imiquimod as ligands with TLR7/TLR9 LBDs using the AutoDock Vina (v.1.1.2.) software.^87^ The docking grid was set using AutoDock Tools (v.1.5.7) software^88^ with a grid of 126 Å × 126 Å × 126 Å to include the entire LBDs of TLR7/TLR9. Polar hydrogens were added to all ligand molecules using AutoDock Tools. The exhaustiveness parameter was set to 20 for docking amino- and carboxy-functionalized NDs, as well as for imiquimod, and to 8 (default value) for the PEGylated NDs. Visualizations of the docked poses and protein structures were performed using PyMOL (v.2.5.5) software (Schrödinger, Inc.).

### Statistical Analysis

At least three individual experiments were performed, using cell lines or primary cells isolated from human donors, and the results are displayed as bar charts with individual data points highlighting mean values and standard deviations. For the CyToF analysis, two-way Anova with Tukey’s comparison was used. For the remaining experiments, one-way Anova with Dunnett’s post hoc test and Student’s *t* test were applied. Significant differences were identified as follows: *p ≤ 0.05, **p ≤ 0.01, ***p ≤ 0.001.

## Supplementary Material











## Notes

Schematic depiction of the study design (Scheme S1) and DLS and XPS results, endotoxin results, flow cytometry results using PBMCs, MDDCs, and CD14-positive monocytes, TEM/SEM imaging of MDDCs, THP-1 cells, and RAW264.7 cells, confocal imaging of MDDCs, HMDMs, and RAW264.7 cells, Western blot analysis of cathepsin B maturation using MDDCs, molecular docking between TLR7/TLR9 and NDs vs imiquimod, the gating strategy applied for CyToF (Figures S1–S20) (PDF), and differentially expressed genes (DEGs) in THP-1 cells:
Control vs ND-NH_2_, ND-COOH, ND-PEG (Table S1) (XLSX)
Control vs ND-NH_2_ (Table S2) (XLSX)
Control vs ND-COOH (Table S3) (XLSX)
Control vs ND-PEG (Table S4) (XLSX)

## ‡‡

Attana AB, 114 19 Stockholm, Sweden

## §§

AstraZeneca, 431 81 Göteborg, Sweden

## ∥∥

Folkhälsan Research Center, 00290 Helsinki, Finland
