# Supplementary material for: Nanodiamonds Interact with Primary Human Macrophages and Dendritic Cells Evoking a Vigorous Interferon Response
Source: ACS Nano. 2025 May 14;19(20):19057–79. doi: 10.1021/acsnano.4c18108 (PMC12120995; doi:10.1021/acsnano.4c18108)
Supplement: Supplementary file 1 [file nn4c18108_si_001.pdf]

Supporting Information

Nanodiamonds Interact with Primary Human Macrophages and Dendritic Cells Evoking a Vigorous Interferon Response

Tomas Malina[1,2,3], Jasreen Kaur[1], Sebastin Martin[1,‡], Audrey Gallud[1,†], Shintaro Katayama[4,§], Arianna Gazzi[5], Marco Orecchioni[6,7], Martin Petr[3], Martin Šrejber[3], Lars Haag[8], Bejan Hamawandi[9], Muhammet S. Toprak[9], Juha Kere[4,10], Lucia Gemma Delogu[11,5], and Bengt Fadeel[1,*]

[1]Institute of Environmental Medicine, Division of Molecular Toxicology, Karolinska Institutet, 171 77 Stockholm, Sweden; [2]Centre for Energy and Environmental Technologies, VSB-Technical University of Ostrava, 708 00 Ostrava, Czech Republic; [3]Regional Centre of Advanced Technologies and Materials, Czech Advanced Technology and Research Institute (CATRIN), Palacký University, 772 00 Olomouc, Czech Republic; [4]Department of Biosciences and Nutrition, Karolinska Institutet, 148 13 Huddinge, Sweden; [5]Department of Biomedical Sciences, University of Padua, Padua 351 29, Italy; [6]La Jolla Institute for Immunology, San Diego, CA 92037, United States; [7]Immunology Center of Georgia, Augusta University, Augusta, GA 30912, United States; [8]Department of Laboratory Medicine, Karolinska Institutet, 141 52 Huddinge, Sweden; [9]Department of Applied Physics, KTH-Royal Institute of Technology, 106 91 Stockholm, Sweden; [10]Stem Cells and Metabolism Research Program (STEMM), University of Helsinki, 00290 Helsinki, Finland; [11]Department of Biological Sciences, Khalifa University of Science and Technology, PO Box 127788, Abu Dhabi, United Arab Emirates. [‡]Present affiliation: Attana AB, 114 19 Stockholm, Sweden; [†]Present affiliation: AstraZeneca, 431 81 Göteborg, Sweden; [§]Present affiliation: Folkhälsan Research Center, 00290 Helsinki, Finland.

*Corresponding author. E-mail: bengt.fadeel@ki.se

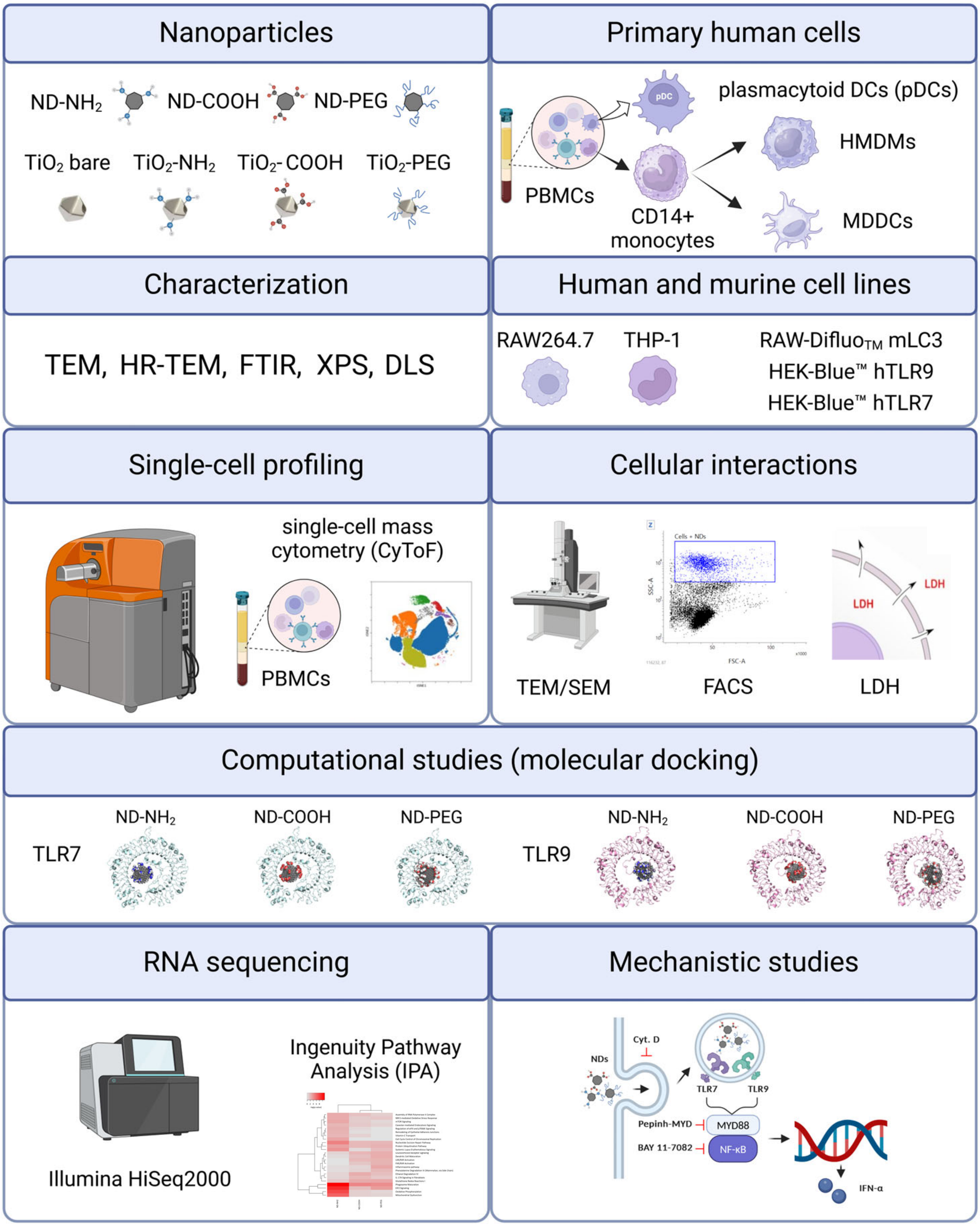

Scheme S1. The study design is schematically depicted. In the present study, amino-, carboxyl- and poly(ethylene glycol) (PEG)-terminated nanodiamonds (NDs) as well as control particles (TiO_2) were evaluated with respect to their impact on immune cells. To this end, peripheral blood mononuclear cells (PBMCs) from healthy adult donors were used. CD14-positive monocytes were isolated from PBMCs and were further differentiated *ex vivo* into macrophages or dendritic cells. PBMCs include lymphocytes (T cells, B cells), natural killer (NK) cells, monocytes, and dendritic cells (DCs) including a rare population of cells known as plasmacytoid DCs. The latter cells were not isolated but were studied *in situ*, as described in the main text. The various experimental and theoretical (*in silico*) methodologies including single-cell mass cytometry applied in the study are illustrated. Mechanistic studies were performed using a combination of pharmacological inhibitors as well as reporter cell lines (stable cell lines labeled with reporter genes). Some elements of the figure were prepared with BioRender.com under an academic license.

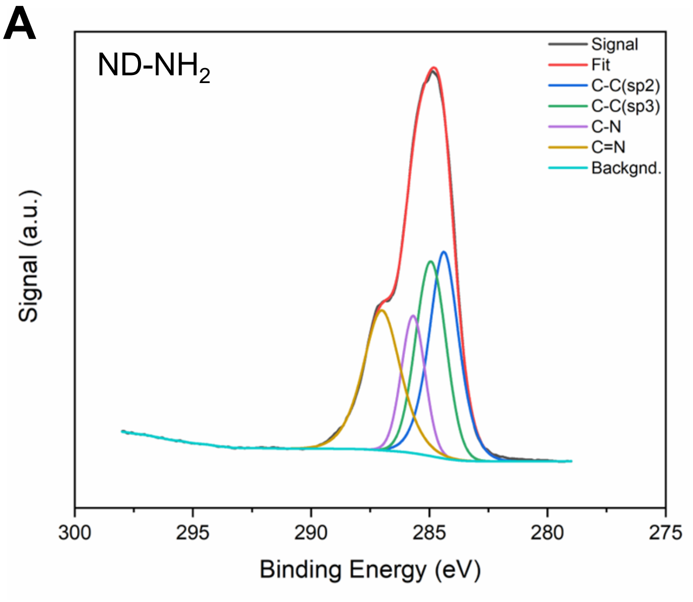

	Atomic %
C1s C-C(sp2)	30.6
C1s C-C(sp3)	27.9
C1s C-N	15.4
C1s C=N	26.2

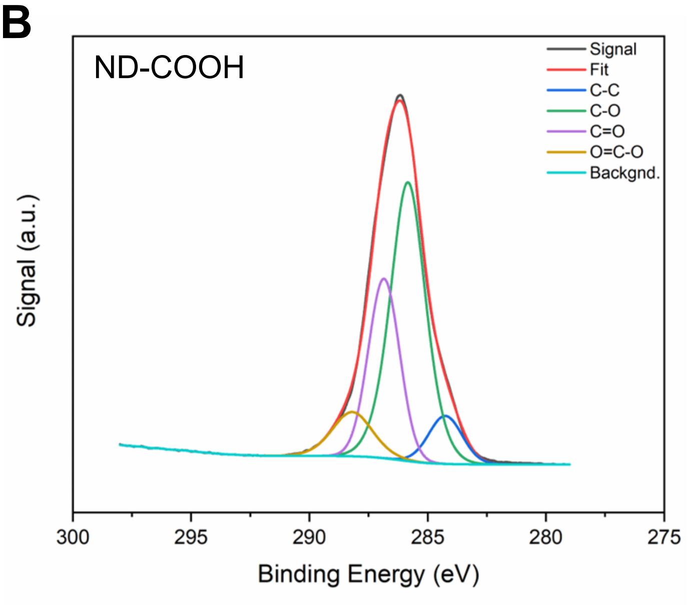

	Atomic %
C1s C-C	8.4
C1s C-O	52.9
C1s C=O	29.2
C1s O=C-O	9.5

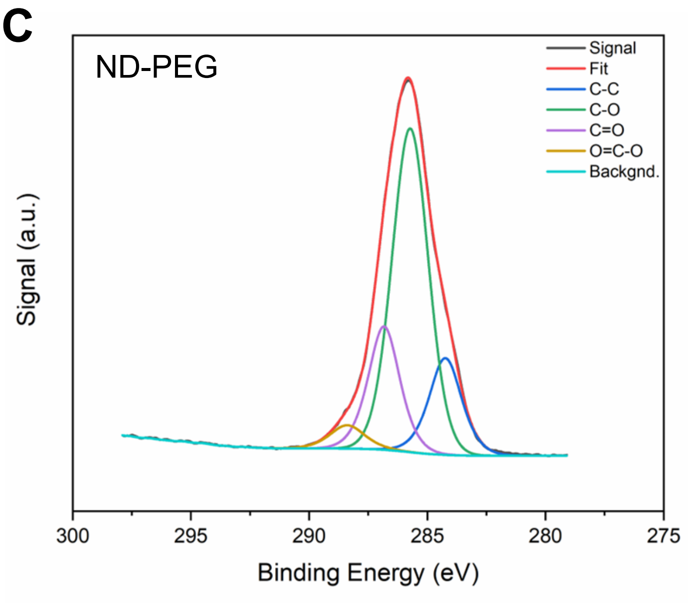

	Atomic %
C1s C-C	16.4
C1s C-O	59
C1s C=O	20.3
C1s O=C-O	4.3

Figure S1. ND characterization. ND-NH_2, ND-COOH, and ND-PEG were analyzed using XPS. High resolution fitted spectra of C 1s of NDs, and atomic composition in % of NDs obtained by XPS.

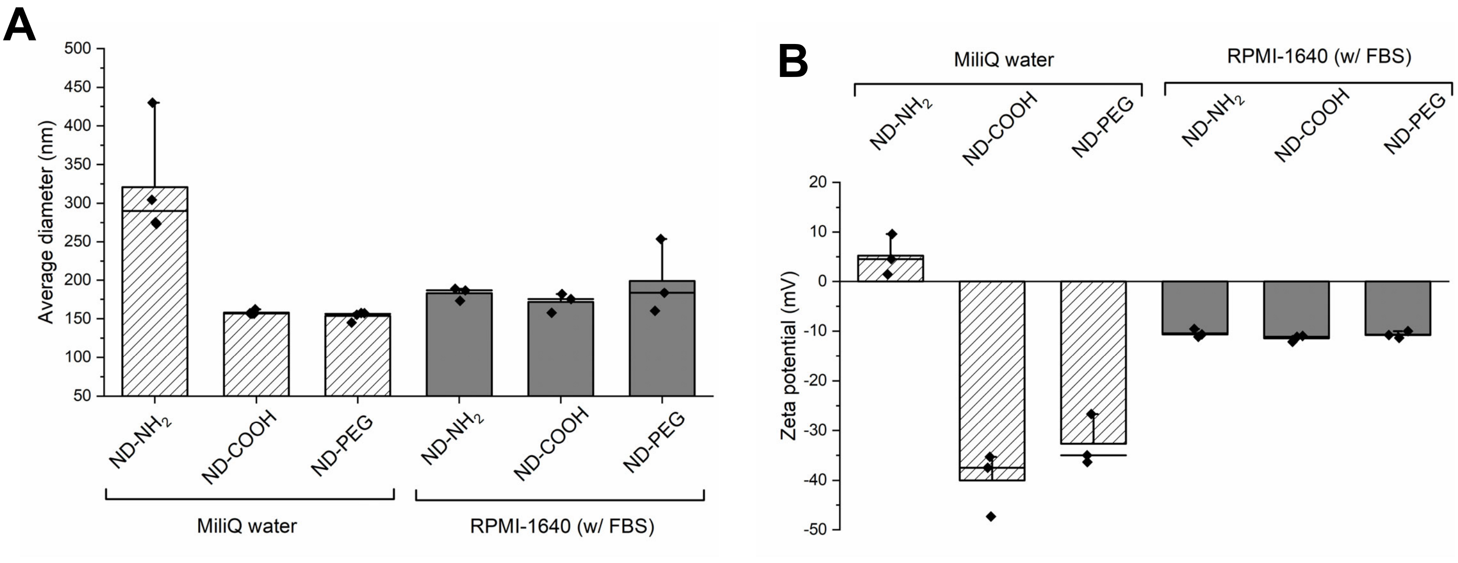

Figure S2. ND characterization. ND-NH_2, ND-COOH, and ND-PEG dispersed in water or in cell culture medium were characterized with respect to hydrodynamic diameter (a) and zeta potential (b).

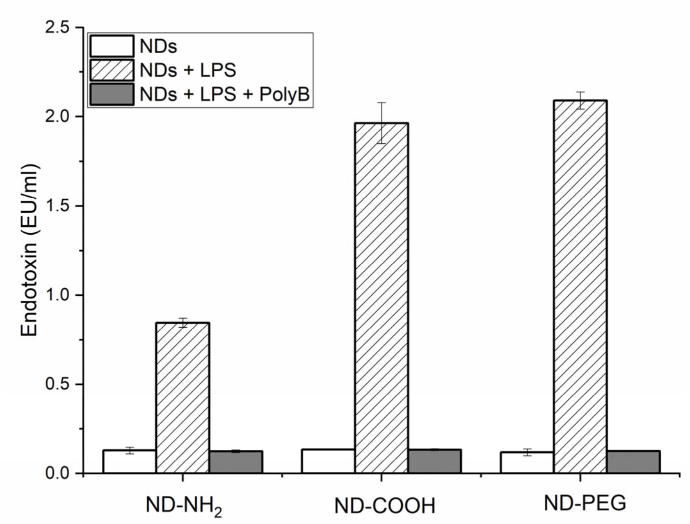

Figure S3. No endotoxin contamination. NDs were tested using the LAL assay. ND samples were spiked with LPS in the absence or presence of polymyxin B to verify the assay.

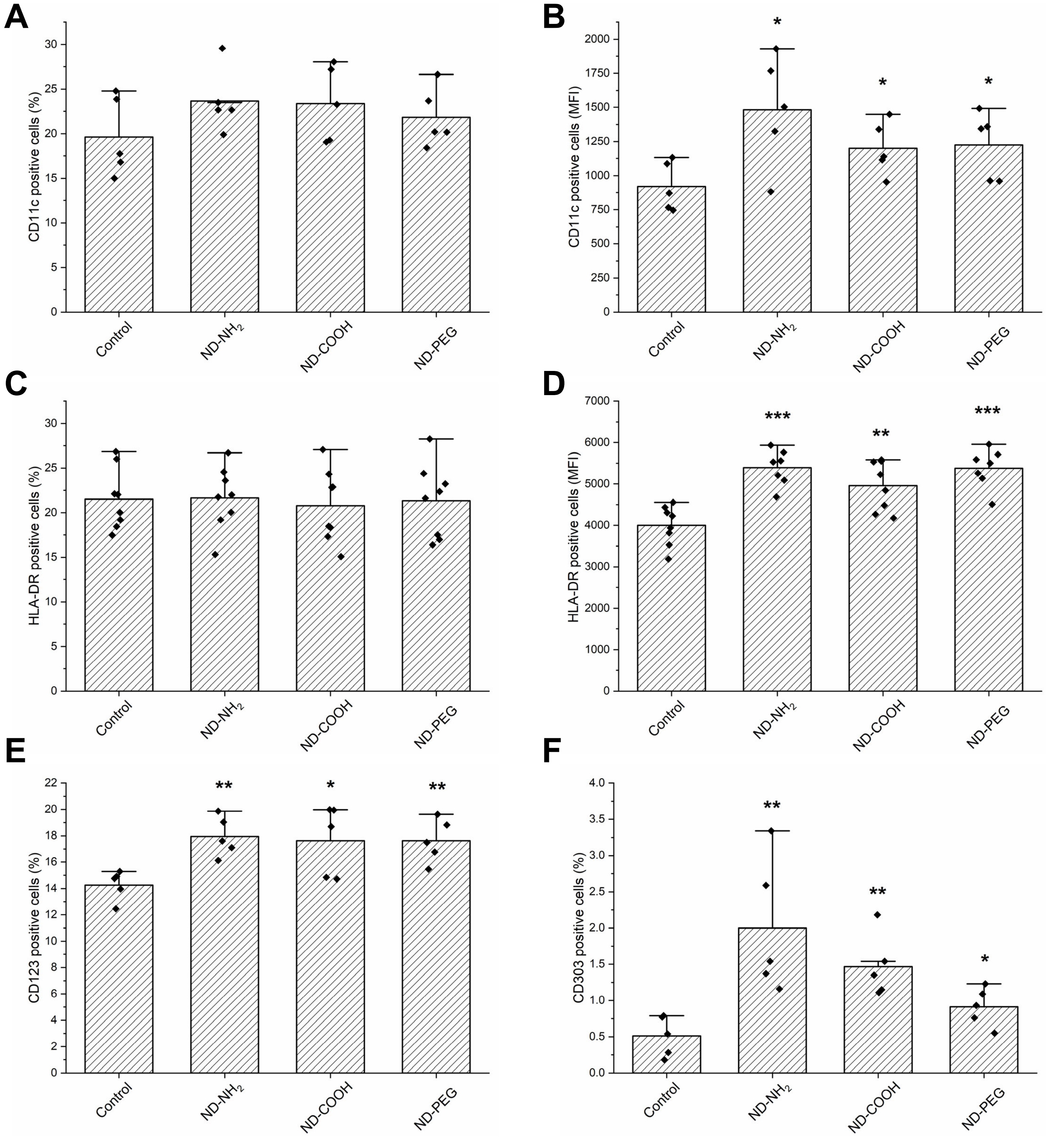

Figure S4. NDs trigger DC maturation. PBMCs were exposed to NDs (25 µg/mL) for 24 h and stained with antibodies against mDC and pDC markers as follows: a) CD11c, c) HLA-DR, e) CD123, and f) CD303. Cells were analyzed by flow cytometry, and the number of positive cells was quantified. For CD11c (b) and HLA-DR (d), the mean fluorescence intensity (MFI) for cells with positive expression of the surface markers was also assessed. Student's t-test was applied. * $p \leq 0.05$, ** $p \leq 0.01$, *** $p \leq 0.001$.

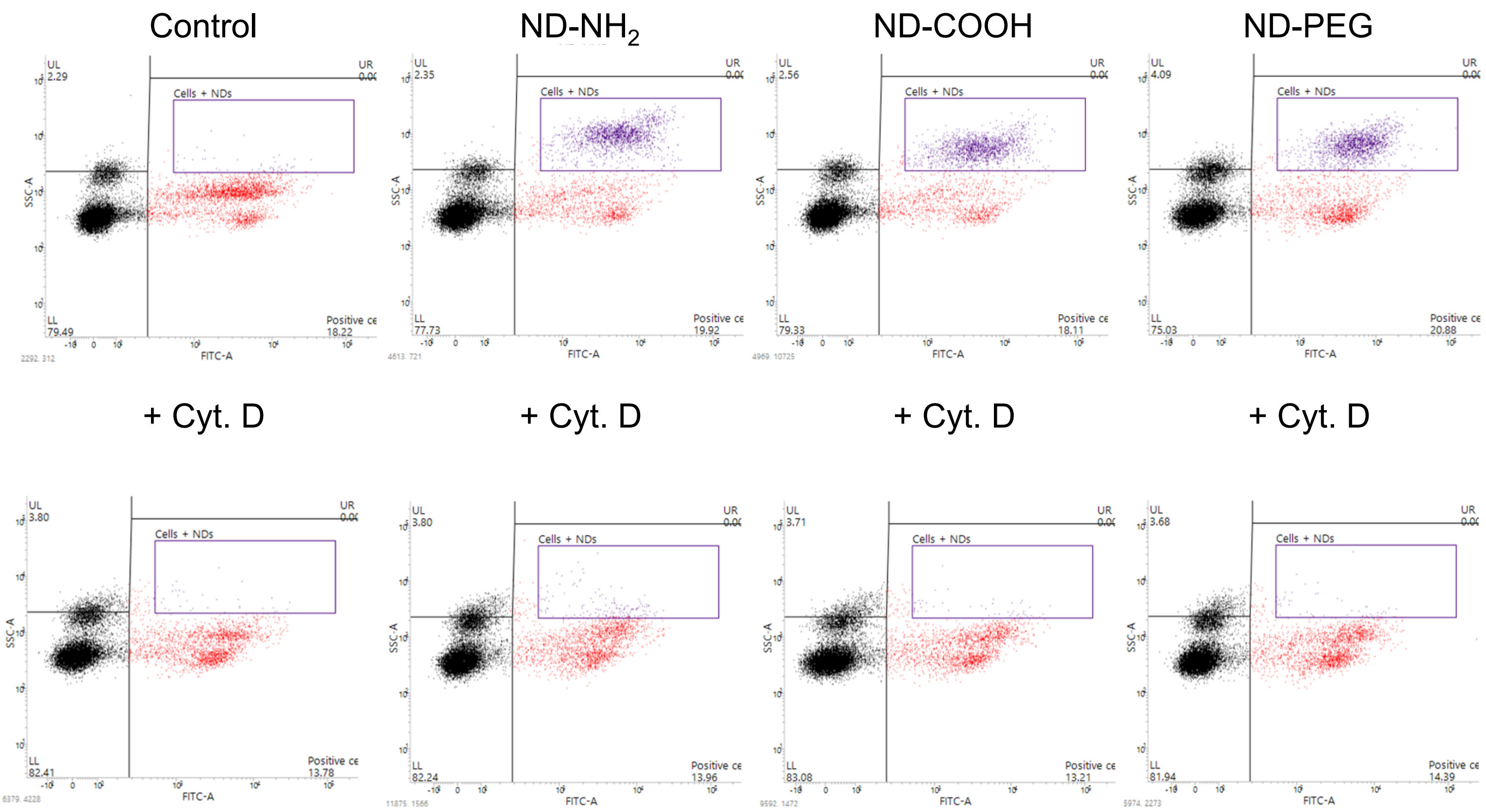

Figure S5. NDs are endocytosed by an HLA-DR-positive subpopulation. PBMCs were exposed to ND-NH_2, ND-COOH, and ND-PEG for 24 h in the presence or absence of cytochalasin D (10 μM) and stained with FITC-labeled mouse anti-human HLA-DR antibodies (BD Biosciences), and the analysis was performed on the gated population as shown. Refer to Figure 4, Figure S6 for more results.

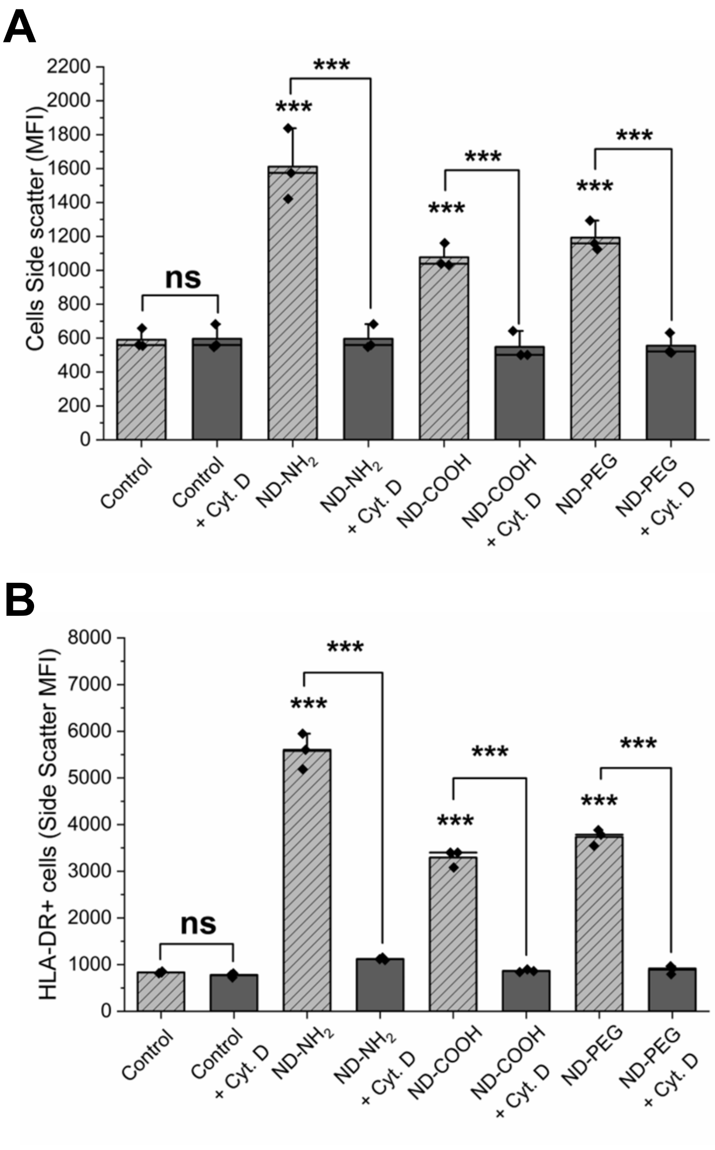

Figure S6. NDs are endocytosed by an HLA-DR-positive subpopulation. (a) PBMCs were exposed to ND-NH_2, ND-COOH, and ND-PEG for 24 h in the presence or absence of cytochalasin D (10 μM). Data are plotted as mean fluorescence values (MFI), which is an indication of the amount of NDs per cell. (b) Cells were stained with antibodies against HLA-DR, and the analysis was performed on the gated population. Data are plotted as mean fluorescence intensity (MFI) values For the % of PBMCs or HLA-DR-positive cells positive for uptake, refer to Figure 4b and c. Results are shown as mean values ± S.D. using cells from three individual donors. Student's *t*-test was applied to determine statistical significance. *** $p<0.001$.

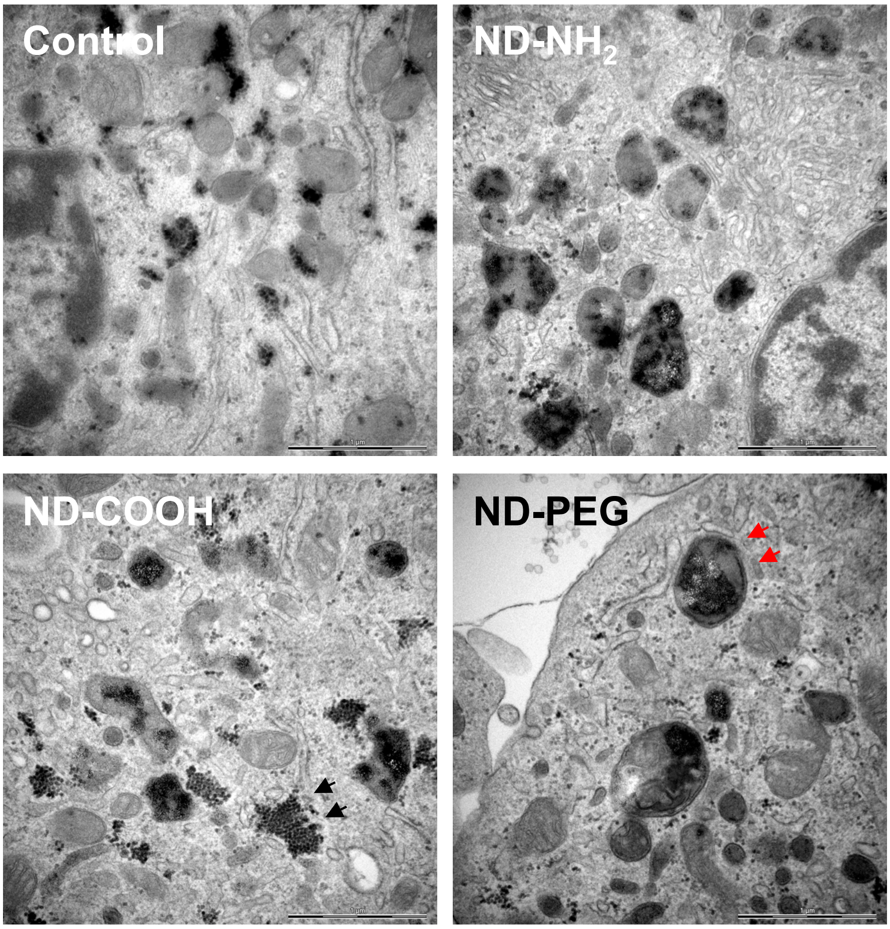

Figure S7. NDs are internalized by MDDCs. TEM images showing primary human monocyte-derived dendritic cells (MDDCs) exposed for 24 h to medium alone (control) *versus* NDs. Clusters of NDs are seen in the cytosol (black arrows), and some of these clusters coincide with electron dense structures, possibly lysosomes (indicated by red arrows). The smaller electron dense dots in the control cell are ribosomes. Scale bars: 1 µm.

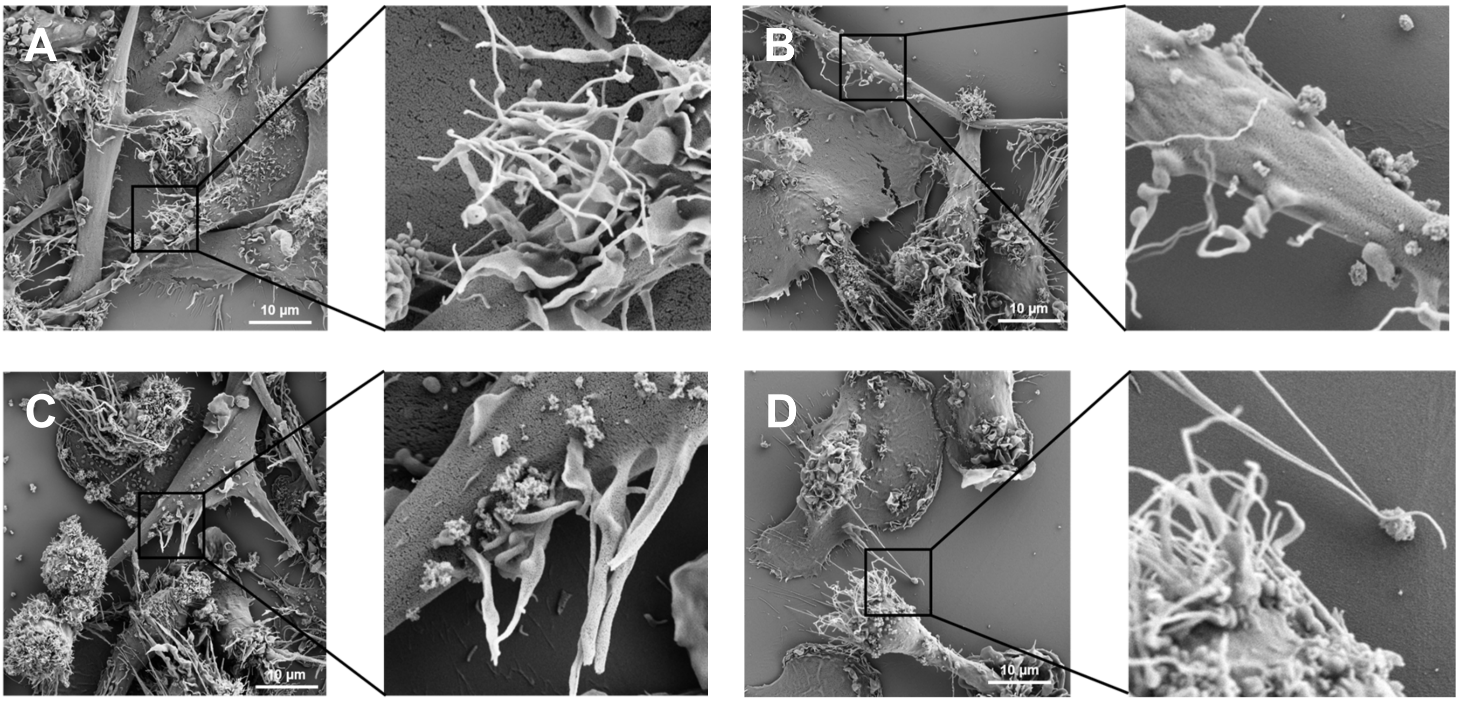

Figure S8. SEM images of MDDCs maintained for 24 h in (A) medium alone (control), or exposed to (B) ND-NH_2, (C) ND-COOH, and (D) ND-PEG at 25 µg/mL in complete cell culture medium. Scale bars: 10 µm.

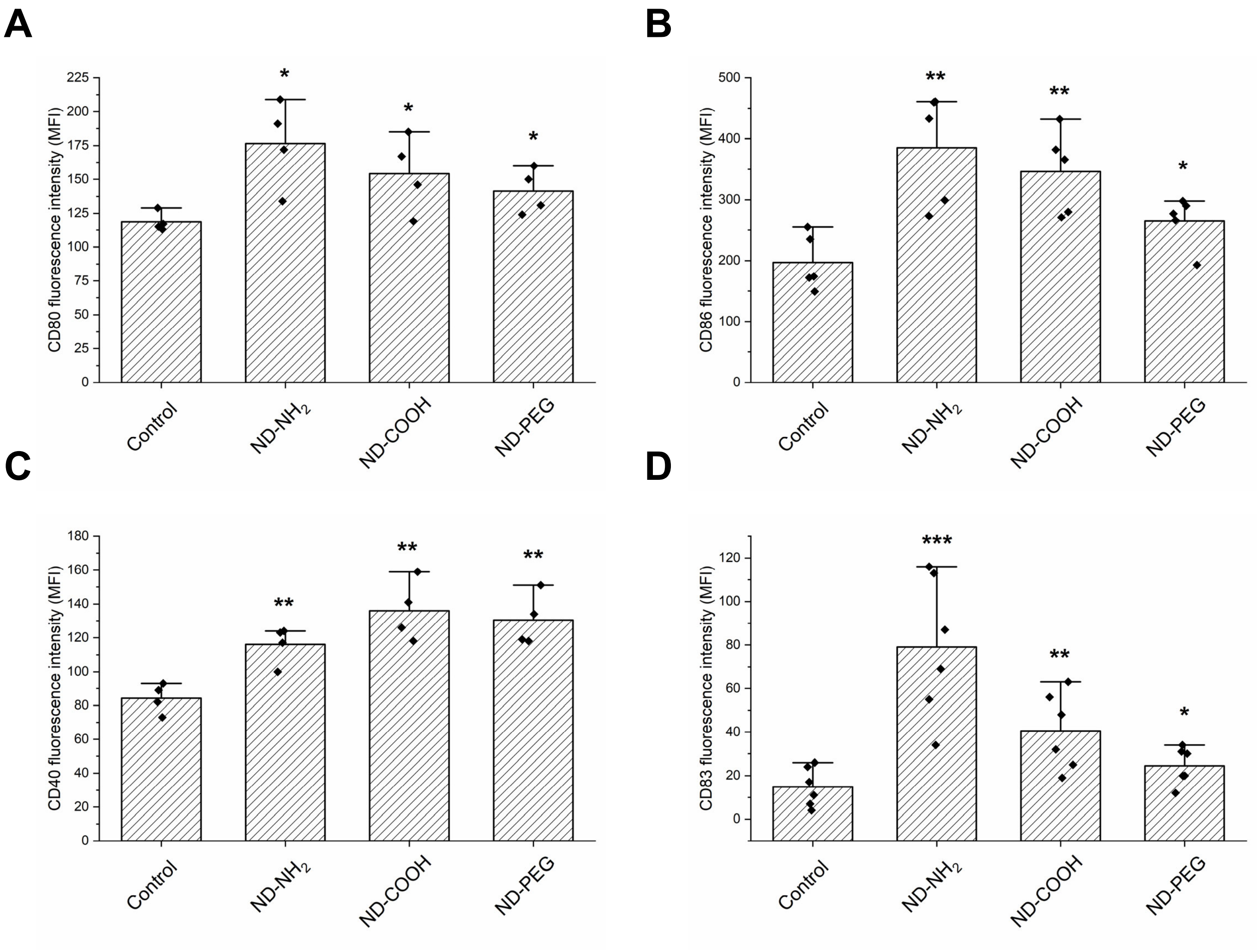

Figure S9. NDs trigger activation/maturation of MDDCs. Cell surface marker expression of activation/maturation markers after 24 h exposure to NDs with varying surface modifications (25 µg/mL). a) CD80, b) CD86, c) CD40, d) CD83. Student's t-test was applied. * $p \leq 0.05$, ** $p \leq 0.01$, *** $p \leq 0.001$.

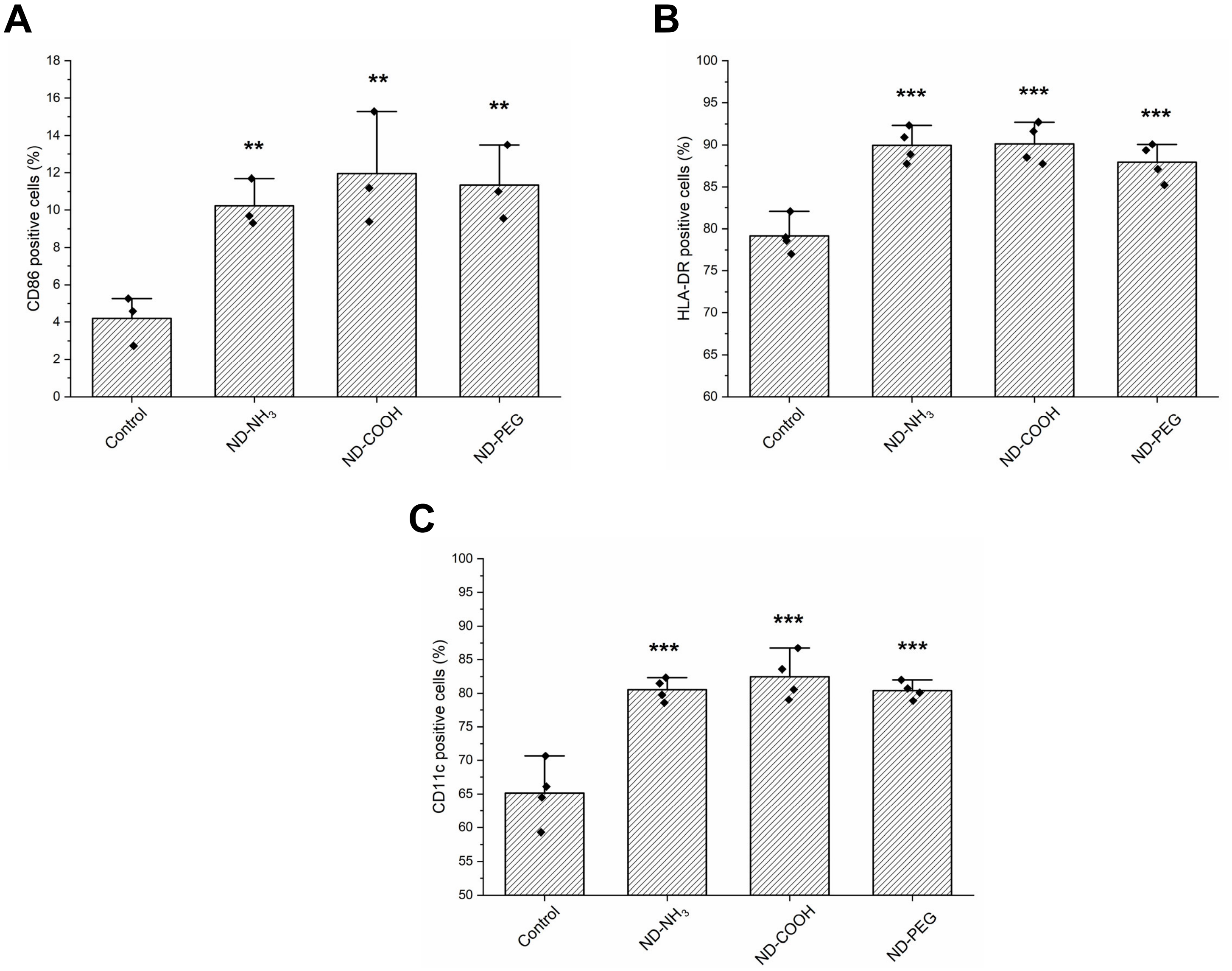

Figure S10. Cell surface markers characteristic of DCs are significantly upregulated in primary human CD14-positive monocytes following the exposure to $ND\text{-}NH_2$, ND-COOH, and ND-PEG for 24 h. Student's t-test was used to evaluate statistically significant differences. ** $p \leq 0.01$, *** $p \leq 0.001$.

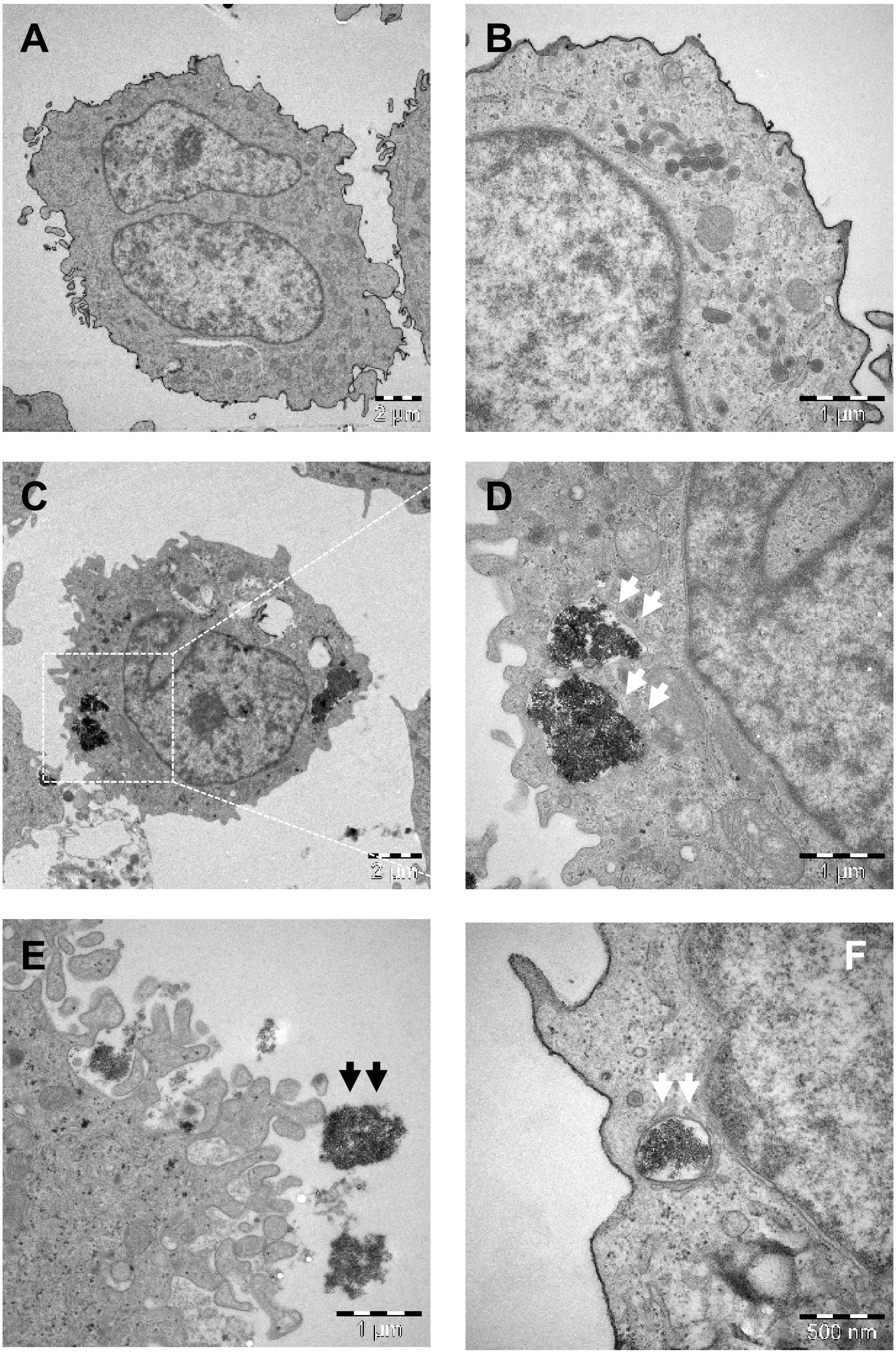

Figure S11. NDs are internalized by THP-1 cells. TEM images showing human monocyte-like THP-1 cells exposed to medium alone (A, B), ND-NH_2 (C, D), ND-COOH (E), and ND-PEG (F) (50 µg/mL) for 4 h. Clusters of NDs are seen within and outside cells. Scale bar: 2 µm (A, C), 1 µm (B, D, E), 500 nm (F).

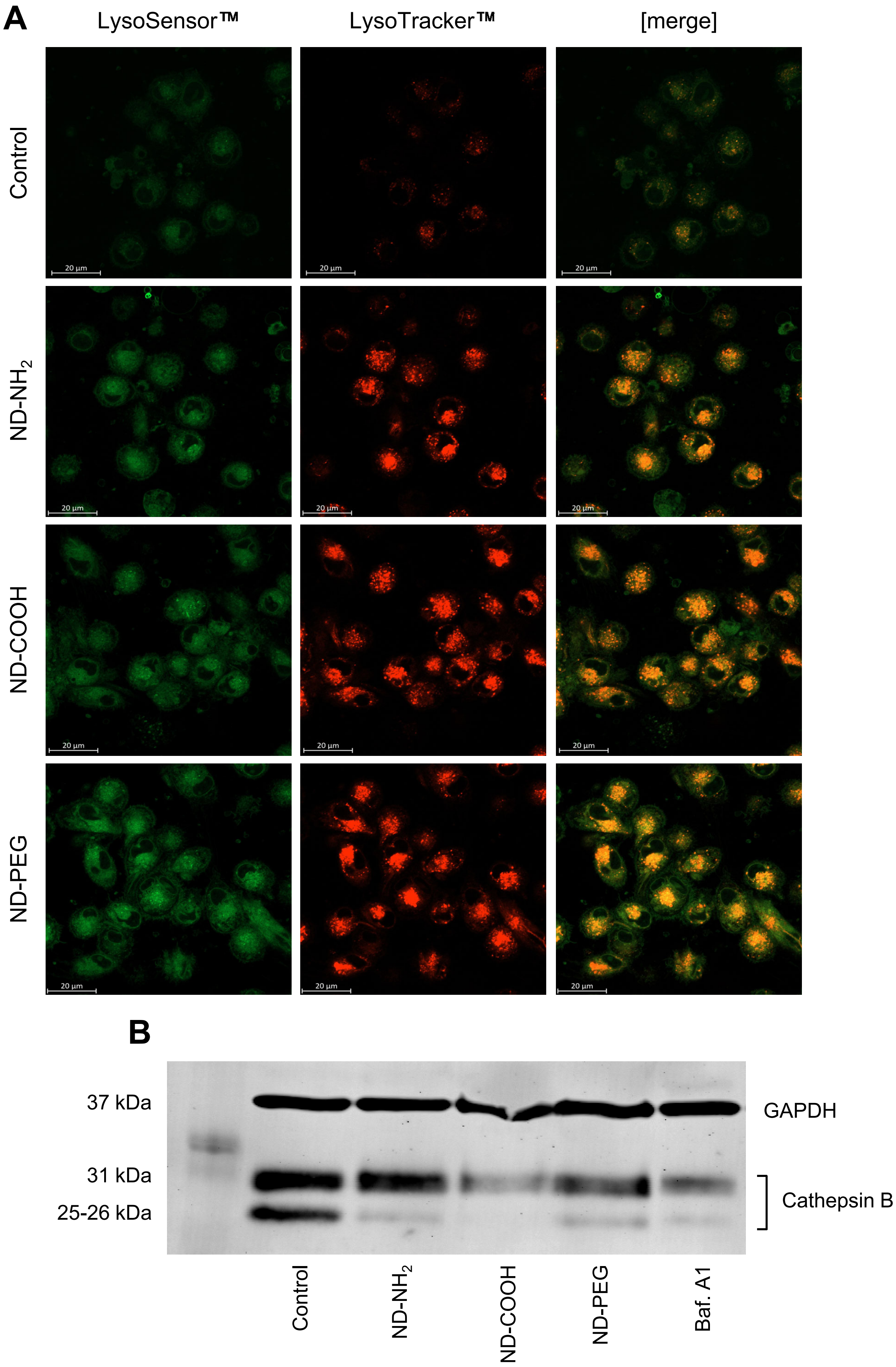

Figure S12. NDs cause lysosomal hyperacidification and impaired maturation of cathepsin B. (a) MDDCs were exposed for 24 h to NDs with different surface modifications (25 µg/mL). Cells were then stained with LysoTracker™ (red) and LysoSensor™ (green) and samples were imaged by confocal microscopy. Scale bars: 20 µm. (b) MDDCs were exposed for 24 h to NDs with different surface modifications (25 µg/mL). Bafilomycin A1 (10 nM) was applied for comparison. Cathepsin B expression and processing (maturation) was monitored by western blot.

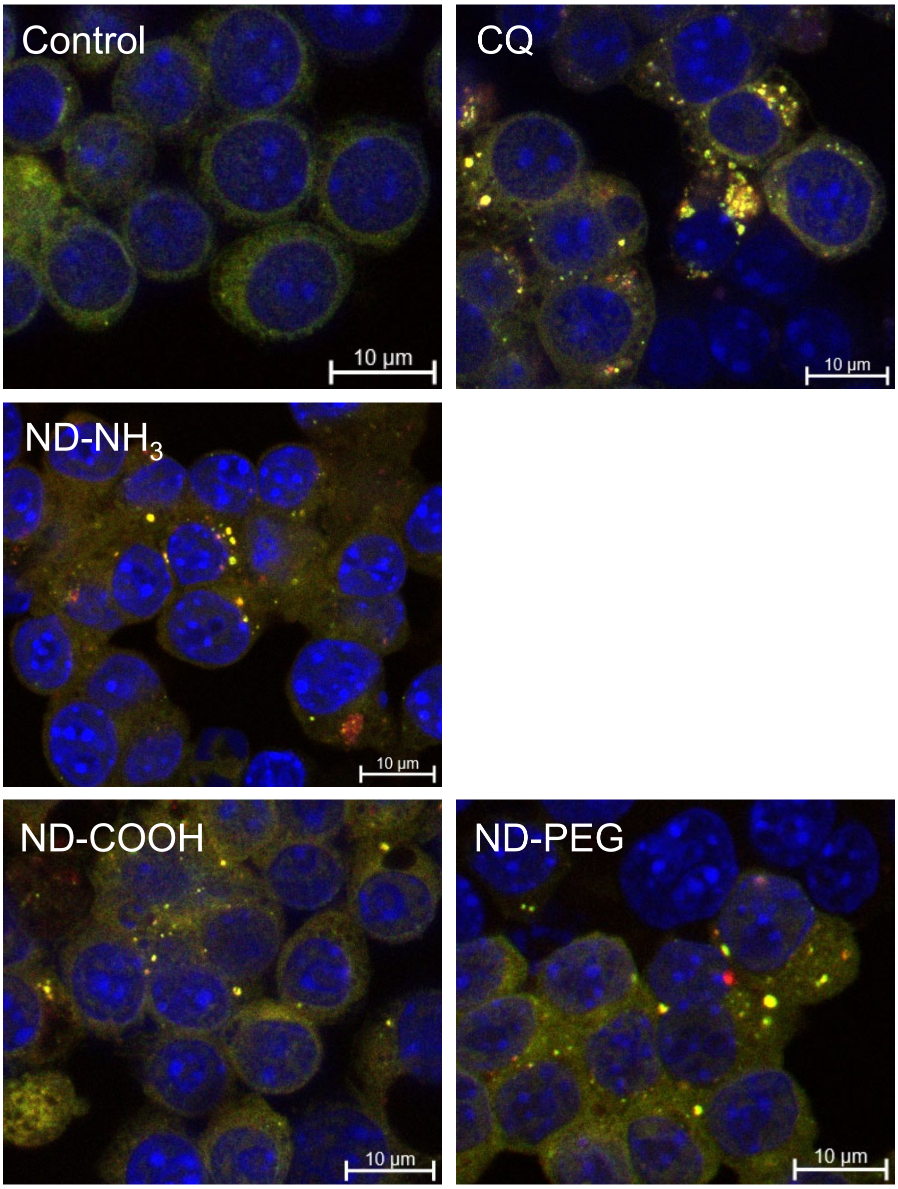

Figure S13. NDs modulate autophagy in a macrophage cell line. Confocal microscopy images of cells exposed to NDs (25 µg/mL) for 24 h. The RAW-Difluo™ mLC3 reporter cell line is engineered to express the RFP::GFP::LC3 fusion protein where LC3B is fused to two fluorescent reporter proteins: RFP (acid-stable) and GFP (acid-sensitive). CQ, chloroquine (100 µM). Scale bars: 10 µm.

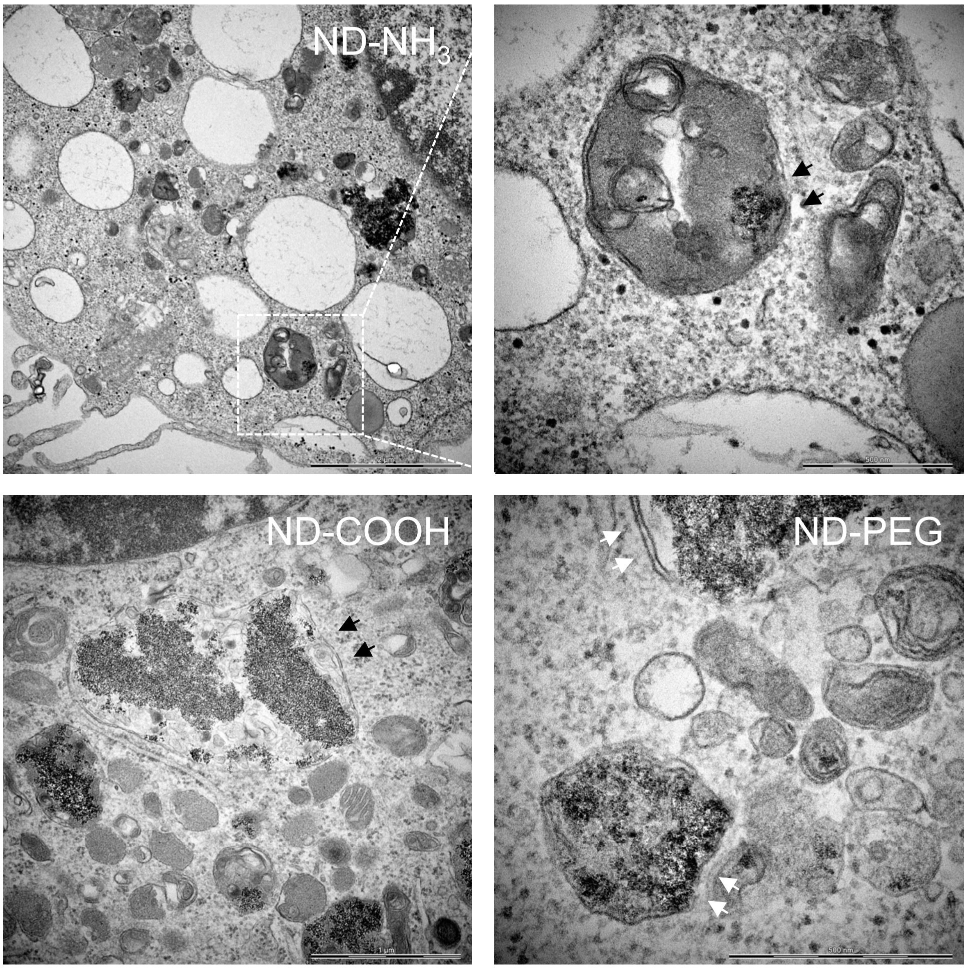

Figure S14. TEM images of RAW264.7 cells exposed to ND-NH$_2$, ND-COOH, and ND-PEG (25 μg/mL) for 24 h. The magnified view shows the presence of NDs within a double-membrane vesicle (autophagosome) (marked with arrows). Scale bars: 2 μm (left) and 500 nm (right) (ND-NH$_2$); 1 μm (ND-COOH); 500 nm (ND-PEG).

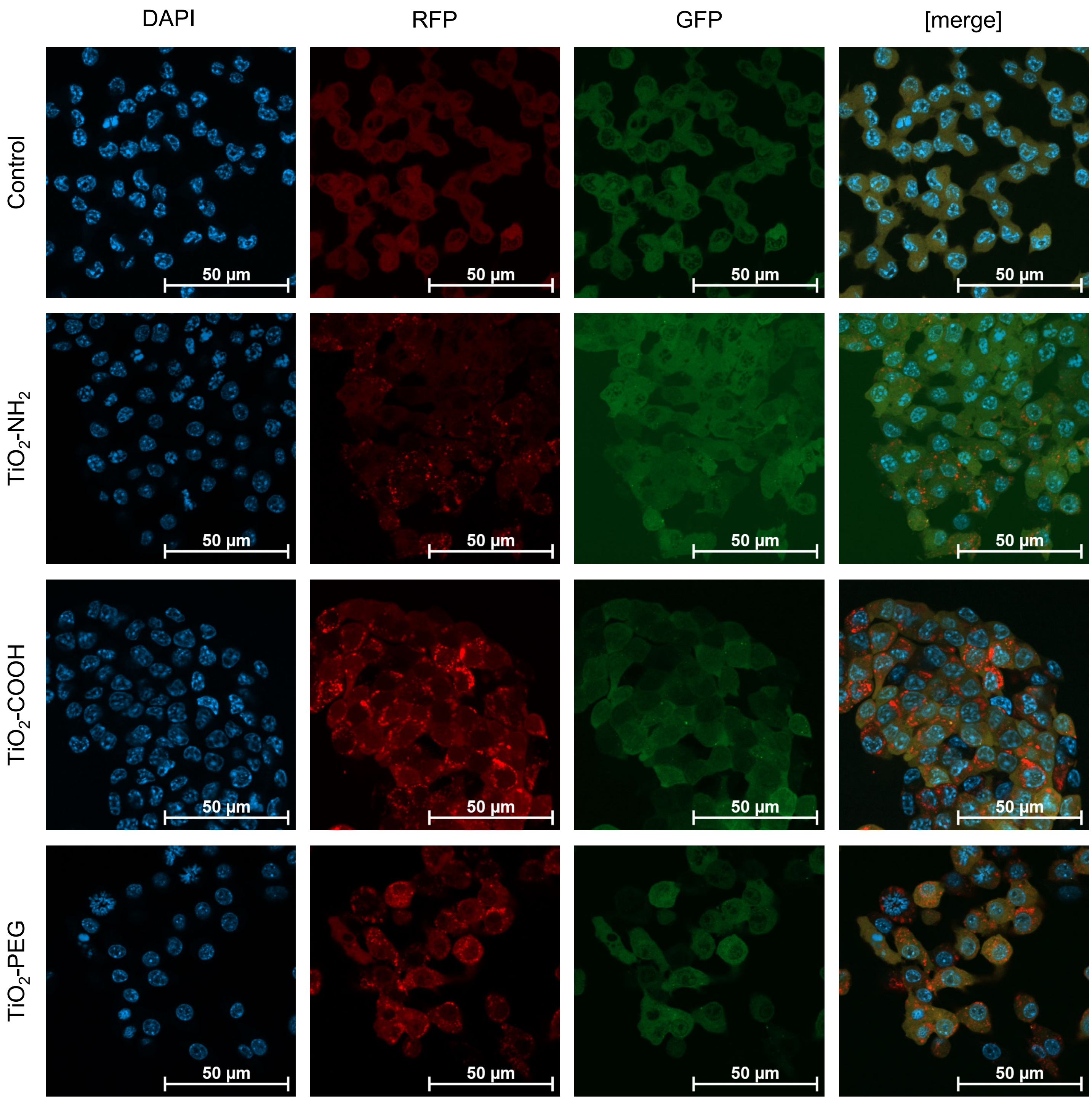

Figure S15. TiO_2 nanoparticles modulate autophagy in a macrophage cell line. Confocal microscopy images of RAW-Difluo™ mLC3 reporter cells exposed for 24 h to TiO_2 nanoparticles with varying surface functionalities (25 µg/mL). Note the absence of green LC3 puncta. Scale bars: 50 µm.

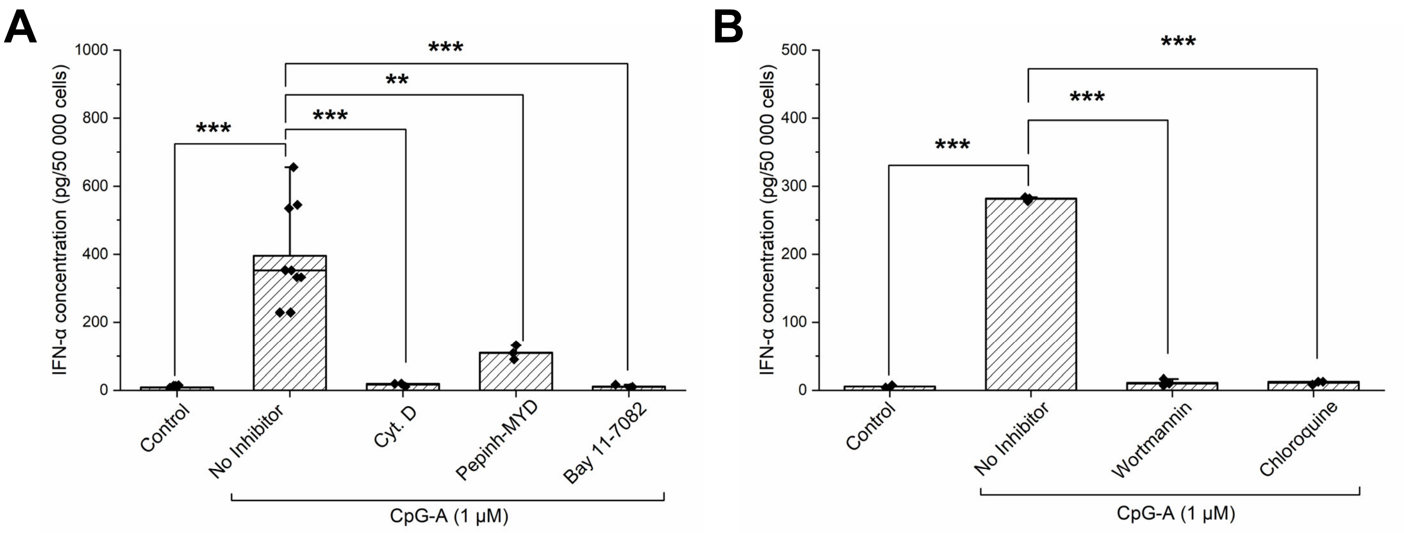

Figure S16. IFN-α production was evaluated in PBMCs exposed for 24 h to the known TLR agonist, CpG-A (1 µM) in the presence or absence of the endocytosis inhibitor cytochalasin D, the MyD88 inhibitor Pepinh-MYD, or the NFκB inhibitor Bay 11-7082 (a), or in the presence or absence of the PI3K inhibitor wortmannin or the autophagy inhibitor chloroquine (b).

A

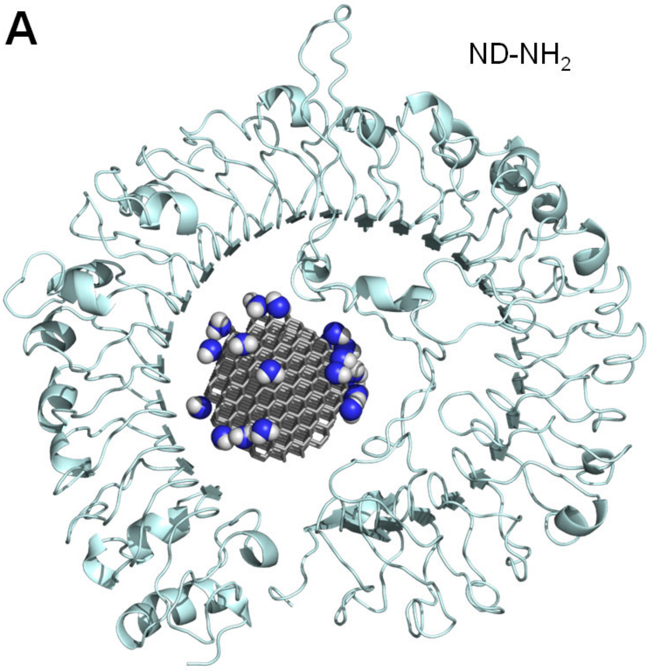

mode	affinity (kcal/mol)	dist from best mode rmsd l.b.	rmsd u.b.
1	-15.6	0.000	0.000
2	-14.1	1.088	12.777
3	-14.0	1.345	9.882
4	-13.8	1.297	6.689
5	-13.8	1.057	10.968
6	-13.7	1.252	9.222
7	-13.7	1.185	12.444
8	-13.5	1.325	10.070
9	-13.5	1.353	11.719

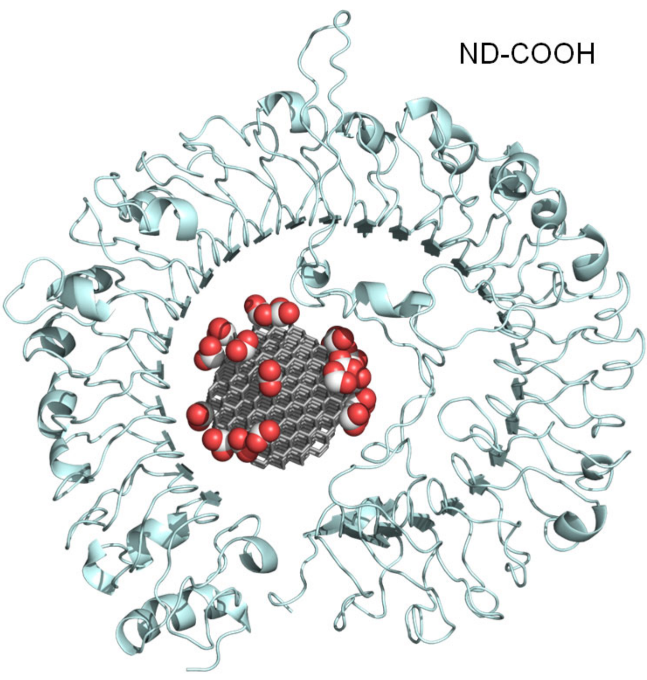

mode	affinity (kcal/mol)	dist from best mode rmsd l.b.	rmsd u.b.
1	-11.7	0.000	0.000
2	-11.4	1.531	11.839
3	-11.2	1.507	12.982
4	-11.2	1.439	6.503
5	-10.8	1.612	10.572
6	-10.6	1.430	11.685
7	-10.5	1.446	12.958
8	-9.8	1.214	3.010
9	-9.7	1.462	13.139

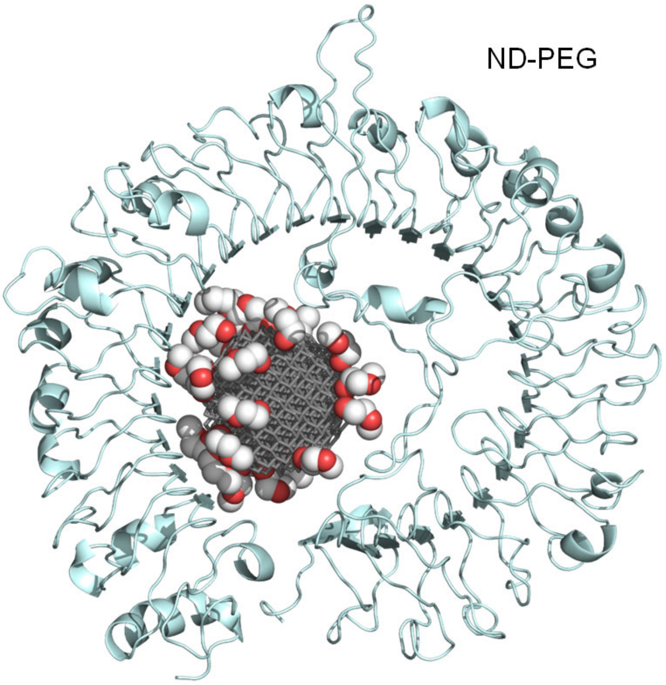

mode	affinity (kcal/mol)	dist from best mode rmsd l.b.	rmsd u.b.
1	-4.7	0.000	0.000
2	-4.6	1.568	13.901
3	-4.5	3.018	12.642
4	-4.4	2.760	14.347
5	-4.3	2.399	11.179
6	-4.3	3.318	14.910
7	-4.3	2.475	13.757
8	-4.2	2.810	8.450
9	-4.2	2.710	12.272

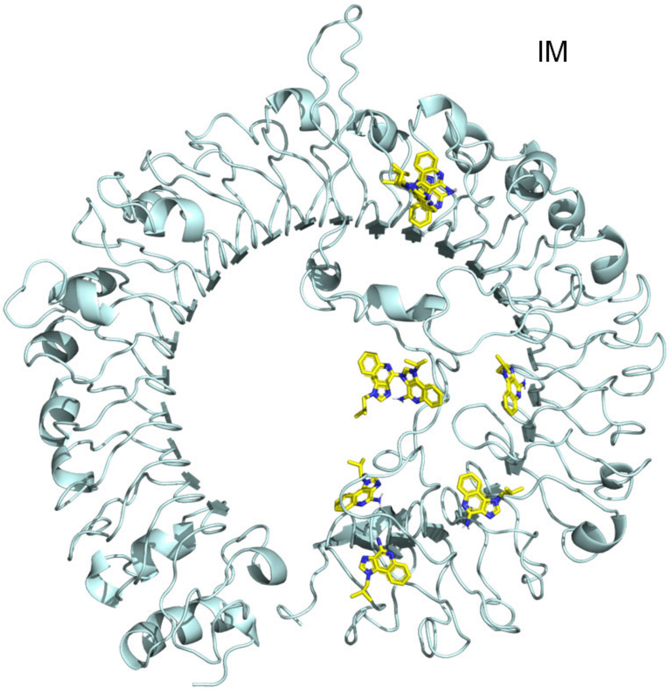

mode	affinity (kcal/mol)	dist from best mode rmsd l.b.	rmsd u.b.
1	-6.3	0.000	0.000
2	-5.8	24.230	27.137
3	-5.6	13.472	15.667
4	-5.3	13.507	14.001
5	-5.2	24.823	27.692
6	-5.1	27.024	29.290
7	-4.9	27.065	29.565
8	-4.9	23.378	24.680
9	-4.8	27.234	28.162

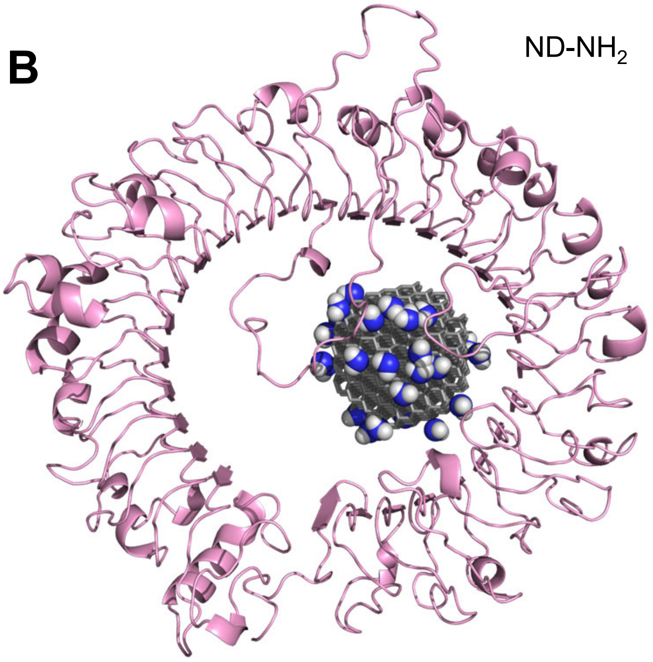

mode	affinity (kcal/mol)	dist from best mode rmsd l.b.	rmsd u.b.
1	-15.6	0.000	0.000
2	-15.5	1.306	11.492
3	-15.5	1.166	12.567
4	-15.5	1.269	10.577
5	-15.5	1.220	12.744
6	-15.5	1.309	11.908
7	-15.5	1.183	10.532
8	-15.2	1.251	11.391
9	-15.1	1.214	12.642

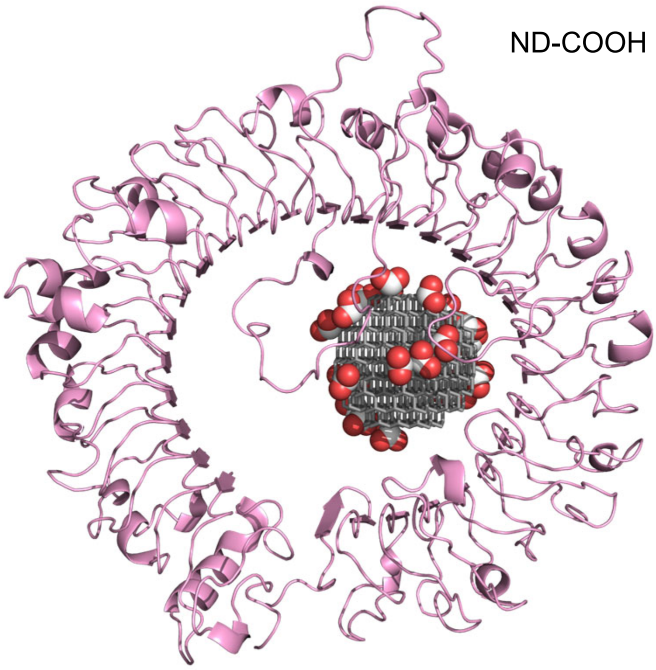

mode	affinity (kcal/mol)	dist from best mode rmsd l.b.	rmsd u.b.
1	-13.0	0.000	0.000
2	-12.8	1.360	10.849
3	-12.8	1.302	13.185
4	-12.4	1.392	4.367
5	-12.3	1.476	12.605
6	-12.3	1.525	13.123
7	-12.2	1.503	12.581
8	-12.2	1.427	9.153
9	-12.2	1.409	9.884

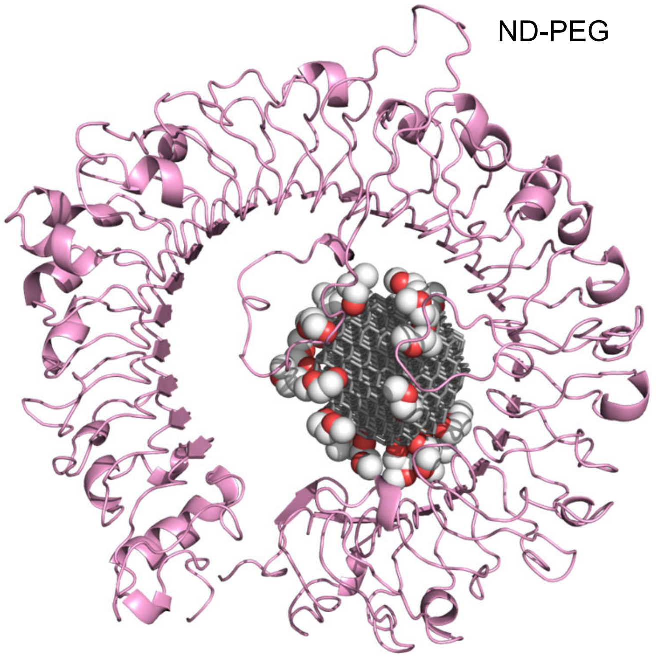

mode	affinity (kcal/mol)	dist from best mode rmsd l.b.	rmsd u.b.
1	-5.0	0.000	0.000
2	-4.9	1.454	3.135
3	-4.8	1.676	13.832
4	-4.8	1.699	9.661
5	-4.7	1.697	9.373
6	-4.7	1.564	3.847
7	-4.6	1.765	9.431
8	-4.6	1.645	4.256
9	-4.5	1.749	13.961

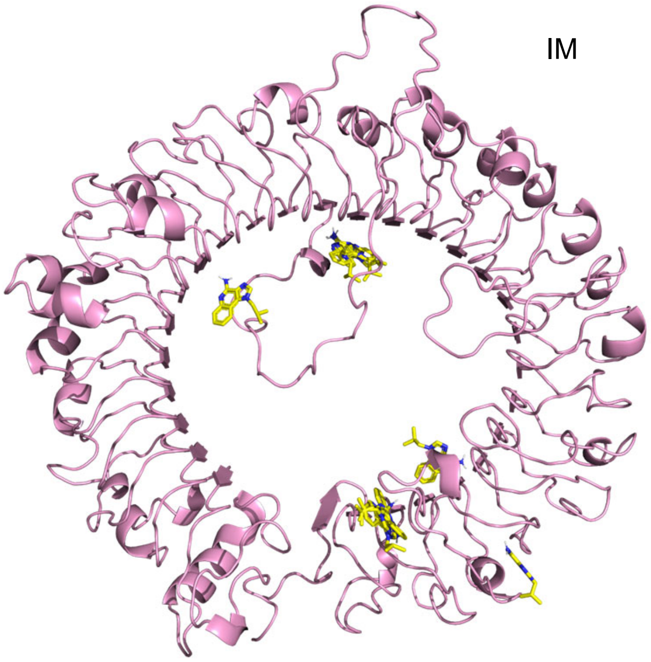

mode	affinity (kcal/mol)	dist from best mode rmsd l.b.	rmsd u.b.
1	-6.8	0.000	0.000
2	-6.8	1.339	1.743
3	-6.2	37.435	39.707
4	-6.1	36.675	38.986
5	-6.1	1.353	4.046
6	-5.9	26.668	29.822
7	-5.7	38.334	40.438
8	-5.5	17.346	19.546
9	-5.4	45.810	48.645

Figure S17. Molecular docking. TLR7 (A) and TLR9 (B) with docking poses for ND-NH_2 (blue), ND-COOH (red), ND-PEG (grey), and imiquimod (yellow). The binding affinities and upper and lower bounds of RMSD with respect to the best binding poses are shown below. For the respective NDs, only the best poses of the docking results are shown for increased clarity while for imiquimod several poses are shown.

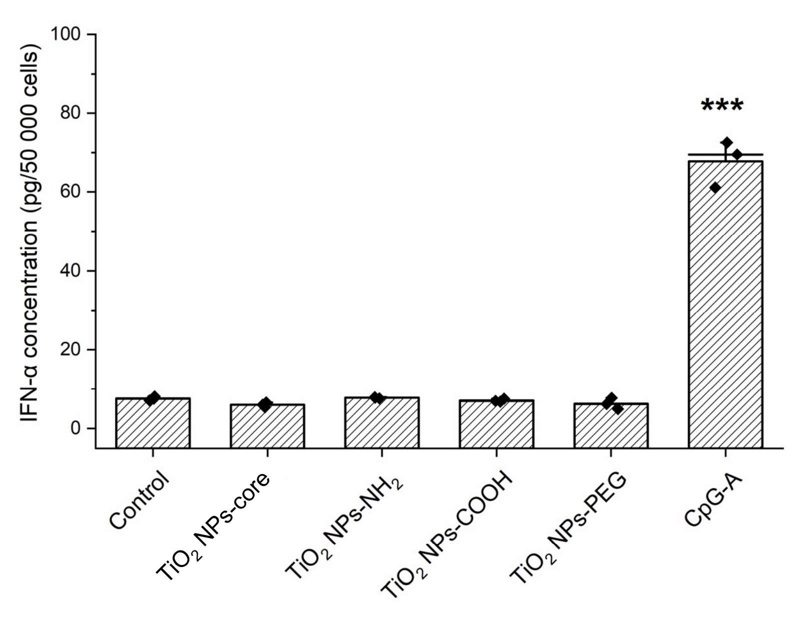

Figure S18. TiO_2 nanoparticles do not trigger a type I interferon response. PBMCs from three human donors were exposed for 24 h to TiO_2 nanoparticles with varying surface functionalities *versus* no surface modification ("core") (50 µg/mL), or to the positive control, CpG-A (1 µM), and IFN-α was detected by ELISA. Data were analyzed using one-way Anova with Dunnett's *post hoc* test. *** $p \leq 0.001$.

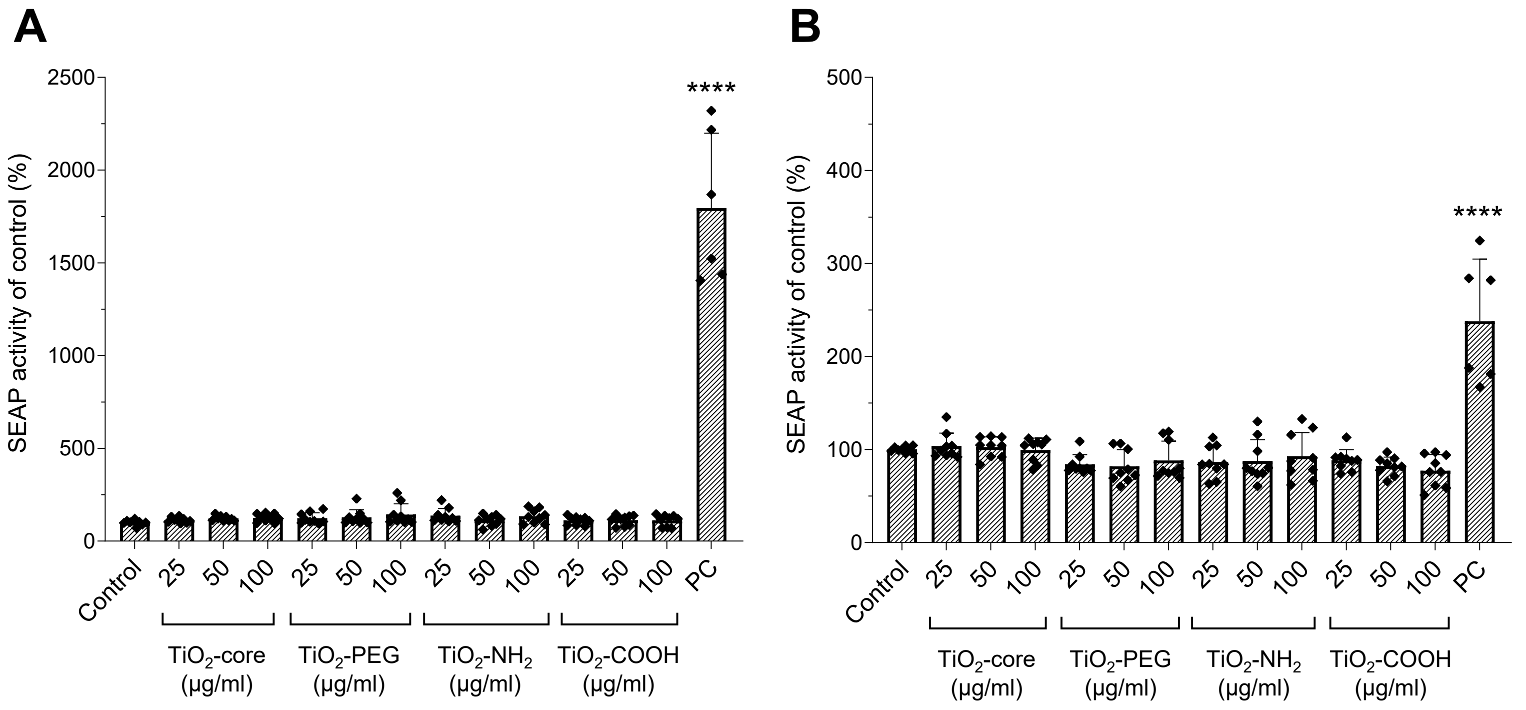

Figure S19. TiO_2 nanoparticles do not activate TLR7 or TLR9. (a) HEK-Blue™ hTLR7 and (b) HEK-Blue™ hTLR9 cells were exposed to TiO_2 nanoparticles at the indicated concentrations for 24 h. PC indicates imiquimod and CpG-A, respectively. Student's t-test was used to assess statistical significance. **** p <0.0001.

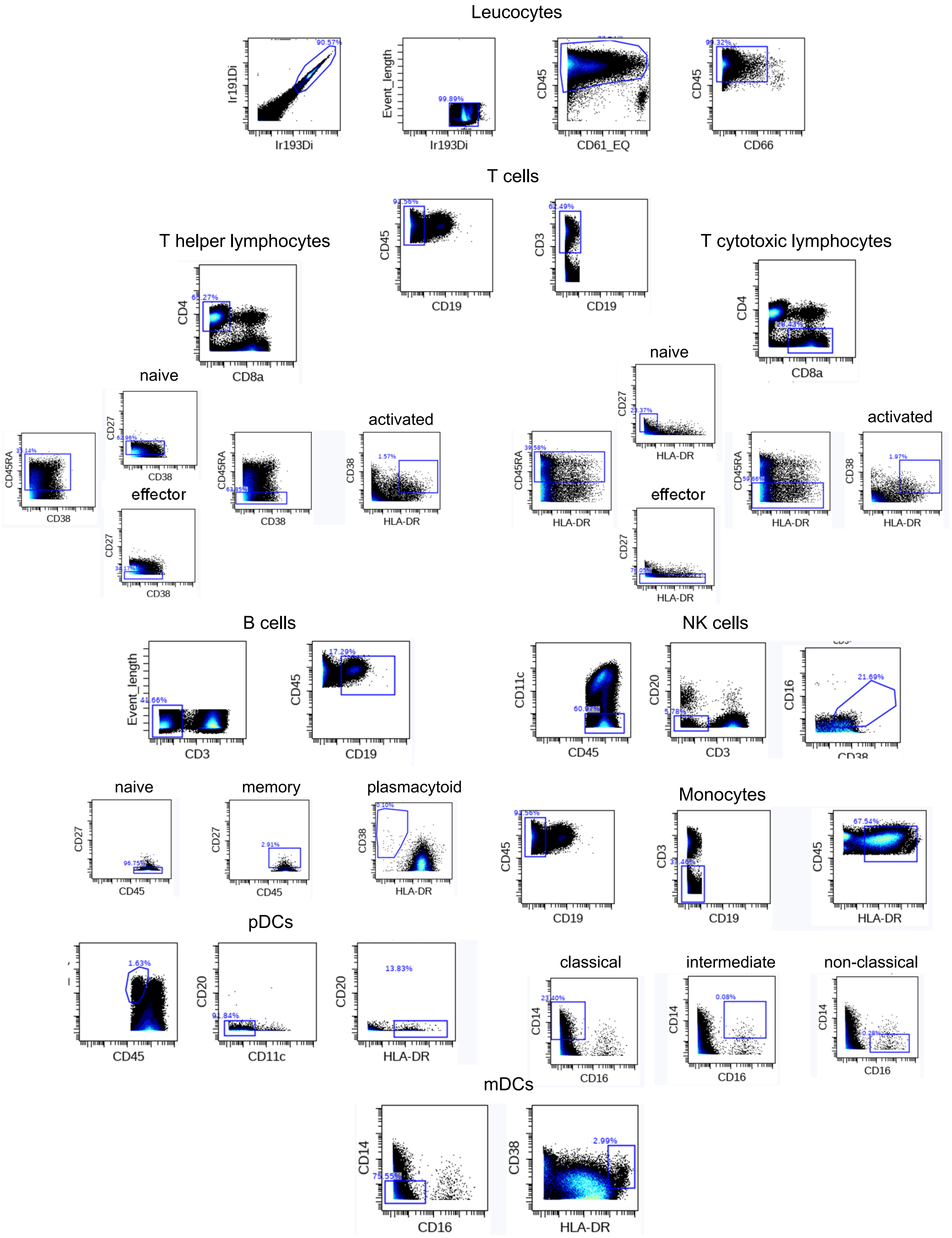

Figure S20. The gating strategy used for the identification of the different immune cell subpopulations.
